# Optical Activity of Spin‐Forbidden Electronic Transitions in Metal Complexes from Time‐Dependent Density Functional Theory with Spin‐Orbit Coupling

**DOI:** 10.1002/open.202200020

**Published:** 2022-05-18

**Authors:** Herbert D. Ludowieg, Monika Srebro‐Hooper, Jeanne Crassous, Jochen Autschbach

**Affiliations:** ^1^ Department of Chemistry University at Buffalo State University of New York Buffalo NY-14260-3000 USA; ^2^ Faculty of Chemistry Jagiellonian University Gronostajowa 2 30-387 Krakow Poland; ^3^ Université Rennes CNRS, ISCR – UMR 6226 35000 Rennes France

**Keywords:** optical activity, spin-forbidden transitions, luminescence, helicenes, time-dependent density functional theory

## Abstract

The calculation of magnetic transition dipole moments and rotatory strengths was implemented at the zeroth‐order regular approximation (ZORA) two‐component relativistic time‐dependent density functional theory (TDDFT) level. The circular dichroism of the spin‐forbidden ligand‐field transitions of tris(ethylenediamine)cobalt(III) computed in this way agrees very well with available measurements. Phosphorescence dissymmetry factors $g_{\mathrm{lum}}$ and the corresponding lifetimes are evaluated for three N‐heterocyclic‐carbene‐based iridium complexes, two of which contain helicene moieties, and for two platinahelicenes. The agreement with experimental data is satisfactory. The calculations reproduce the signs and order of magnitude of $g_{\mathrm{lum}}$ , and the large variations of phosphorescence lifetimes among the systems. The electron spin contribution to the magnetic transition dipole moment is shown to be important in all of the computations.

## Graphical abstract

**Circularly polarized luminescence** (CPL) dissymmetry factors and phosphorescence lifetimes are calculated for the spin‐forbidden triplet emission of metal‐helicene complexes. Spin‐forbidden singlet‐triplet excitations and electronic circular dichroism are calculated for tris(ethylenediamine)cobalt(III). The results agree well with reported experimental data. Calculations are based on two‐component relativistic time‐dependent density functional response including the effects from spin‐orbit coupling.

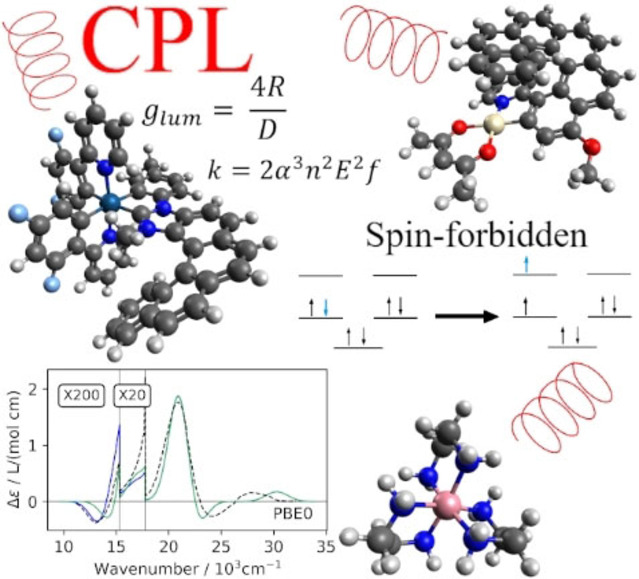

## Introduction

There is continuing strong interest in the photophysical properties of metal complexes, driven by numerous applications such as electro‐emissive switches, luminescent sensors, cellular imaging agents, photosensitizers, and emissive materials for organic light‐emitting diode (OLED) technology. Among phosphorescent emitters, Ir(III) compounds with a pseudo‐octahedral coordination environment and Pt(II) systems with square‐planar structures have emerged as particularly appealing and thus have been developed and examined extensively.[^1^, ^2^, ^3^, ^4^] For example, Ir(ppy)_3_ (ppy=2‐phenylpyridine) has been studied intensely by theory and experiment in the context of developing complexes with strong emission in different parts of the visible spectrum.[^5^, ^6^, ^7^, ^8^, ^9^, ^10^, ^11^, ^12^, ^13^, ^14^, ^15^]

**Figure 1:**
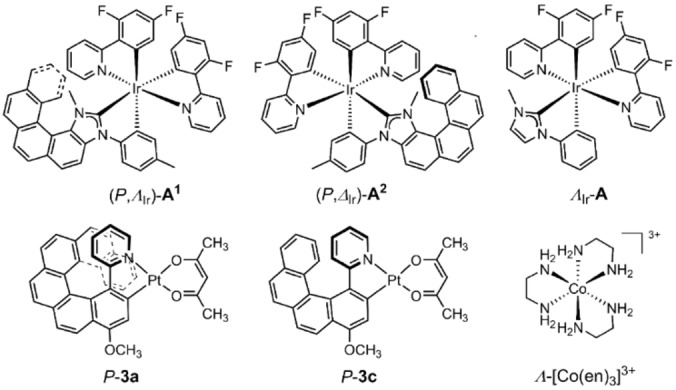
Metal complexes studied in this work.

Chiral enantiopure systems give access not only to desired emission wavelengths, but also the ability to modulate circularly polarized (CP) luminescence (CPL).[^16^, ^17^, ^18^, ^19^] Strong optical activity of metal complexes can be obtained, in particular, when not only the relative arrangement of the ligands is chiral, but when the ligands themselves are inherently chiral and display strong optical activity themselves. Helicenes play a particularly important role in this context.[^20^, ^21^, ^22^] For example, chiral Ir(III) complexes with a pentahelicenic N‐heterocyclic carbene (NHC) ligand such as (*P*,Λ_Ir_)‐**A** ^1^ and (*P*,Δ_Ir_)‐**A** ^2^ (Figure 1; note that the systems are diastereoisomers differing in absolute configuration at the metal center) reported in Ref. [23] have shown promise as chiral dopants in CP‐OLEDs and singlet‐oxygen sensitizers, among other applications,[^17^, ^24^, ^25^, ^26^] because they displayed very long‐lived CP blue‐green phosphorescence. The emission lifetimes were increased by a factor of 100, and the sign of the CPL signal was controlled by the stereochemistry of the π‐helical NHC ligand, in comparison to the non‐helicenic NHC model complex Λ_Ir_‐**A**. Another very interesting class of chiral systems combining metal centers and helicene moieties are platinahelicenes.[^27^, ^28^, ^29^, ^30^, ^31^, ^32^] Chemical formulas for two representative systems from Ref. [30], platina[8]helicene *P*‐**3 a** and platina[6]helicene *P*‐**3 c**, are shown in Figure 1. Here, the metal atom is part of the helical π‐system of the chromophore, leading to room‐temperature CP phosphorescence, and useful redox activity, among other desirable properties. Importantly, *P*‐**3 c** was utilized to prepare the first helicene‐based CP‐OLED shown to yield high electrophosphorescence.^[17]^

Long‐lived phosphorescence is associated with spin‐forbidden radiative electronic transitions to the ground state. Spin‐orbit coupling (SOC), a relativistic effect^[33]^ generated in the electronic structure by the presence of the high nuclear charge of the heavy metal, is responsible for lifting the spin selection rule, such that these transitions become weakly allowed. For the rational development of CP emitters, it is very important that their physico‐chemical properties can be predicted and analyzed with the help of quantum‐chemical theoretical methods. The most important parameters are the oscillator or dipole strength (f or D ), and the phosphorescence lifetime τ or rate k=1/τ for the spin‐forbidden transition. For CP emitters, in addition to the phosphorescence quantum yields and lifetimes, the rotatory strength R and the luminescence dissymmetry factor $g_{\mathrm{lum}}=2\left(I_{L}-I_{R}\right)/\left(I_{L}+I_{R}\right)$ are the key parameters pertaining to the optical activity of the emission.

A popular wavefunction theory approach for calculating excited‐state properties and transition moments is based on the complete active space (CAS) self‐consistent field (SCF) method and its variants and extensions,^[34]^ with treatment of SOC via state interaction, as implemented, for example, in the Orca^[35]^ and Molcas programs.^[36]^ Such calculations have been performed in the context of the optical activity of metal complexes such as *P*‐**3 c** and the *D*_3_ symmetric benchmark systems [Co(en)_3_]^3+^ and tris(DPA)europium(III) (en=ethylenediamine=1,2‐diaminoethane, DPA=(2,6)‐pyridinedicarboxylate).[^30^, ^37^] However, some difficulties were noted regarding the size of the active space required to obtain reliable intensities, and the scaling of CASSCF‐based methods with the system size and active space is unfavorable. A less demanding correlated wavefunction approach is the approximate coupled‐cluster with singles and doubles method called CC2, for which CP luminescence calculations for organic molecules have been reported.^[38]^ For larger systems, time‐dependent density functional theory (TDDFT), in particular in its response framework, remains the method of choice for most practical applications in photochemistry,[^39^, ^40^, ^41^, ^42^] including optical activity[^43^, ^44^] and specifically including CP luminescence.[^45^, ^46^] With certain approximations and simplifications,[^47^, ^48^] even very large molecules and molecular aggregates can be targeted. A recent study^[49]^ reported a series of calculations of the spin‐allowed vibronic electronic circular dichroism (ECD) and one‐photon vibrationally resolved absorption and emission spectra of the iridium complexes of Figure 1, underlining the interest in such systems as well as pointing out the lack of reliable relativistic methods for spin‐forbidden CP computations for these types of compounds. Spin‐forbidden CP emission of some organic molecules was determined in Ref. [50] from TDDFT calculations that treated SOC as a linear perturbation. For systems with heavy atoms such as the ones shown in Figure 1, this level of treating SOC is unlikely to be sufficient. For example, inspection of the formalism does not indicate that there are electron spin contributions to the magnetic transition dipole moments entering the rotatory strengths. Two‐component relativistic TDDFT (2c‐TDDFT) approaches for treating spin‐forbidden transitions and phosphorescence lifetimes with SOC included variationally are also available, and were demonstrated to be applicable to complexes with heavy metals of the size that would be of interest in CP‐OLED applications.[^11^, ^51^, ^52^, ^53^] In particular, Mori et al.^[10]^ demonstrated applicability of the method of Ref. [51] to calculate phosphorescence lifetimes and the T_1_ state zero‐field splitting for a large variety of organometallic complexes.

For the purpose of studying the photophysical properties, and specifically the CP luminescence, of heavy metal complexes, we extended the efficient 2c‐TDDFT code used by Mori et al.,^[10]^ to calculate also the rotatory strengths of the transitions, a feature not previously available in this program. We then applied it to the systems shown in Figure 1. These complexes have been characterized experimentally previously, and TDDFT calculations of the spin‐allowed ECD were shown to match well with measured data.[^23^, ^27^, ^28^, ^30^] However, it was not possible to support the experimental characterizations of the CP phosphorescence fully by calculations. The present study aims to fill this gap. In particular, we show that the calculated $g_{\mathrm{lum}}$ of *P*‐**3 c** is consistent with a revised experimental value that differs considerably from the one reported in Ref. [30]. Furthermore, electron spin contributions to the magnetic transition moments are shown to be responsible for the positive signs of $g_{\mathrm{lum}}$ of complexes *P*‐**3 a** and *P*‐**3 c**. In addition to the emission data of the Ir and Pt complexes in Figure 1, we also investigate the ECD of the ligand‐field (LF) transitions of tris(ethylenediamine)cobalt(III) (Λ‐[Co(en)_3_]^3+^). An experimental spectrum, including the weakly intense triplet excitations at low energy, is available,^[54]^ and spin‐allowed natural electronic optical activity of the complex has been studied extensively in the past using TDDFT methods,[^55^, ^56^, ^57^] rendering it a suitable system to benchmark new theoretical methods for the optical activity of metal complexes.

## Theory

The formalism outlined in the following was implemented in the 2019 version of the Amsterdam Density Functional (ADF) program,^[58]^ and in a 2021 developer's version of ADF. All‐electron relativistic computations were based on the two‐component zeroth‐order regular approximation (ZORA) Hamiltonian^[59]^ and Slater‐type orbital (STO) basis sets optimized for ZORA calculations,^[60]^ as specified in the Computational Section. Excitation energies and transition moments were computed with the ‘Excitations’ module of ADF.[^51^, ^61^] The calculation of rotatory strengths of spin‐allowed transitions, using the dipole‐length and dipole‐velocity gauges, with the latter being origin invariant by construction, was previously implemented in ADF by one of us.^[55]^ For the present study, the functionality of the 2c‐TDDFT module in ADF was extended in a similar way to determine the dipole‐velocity in addition to the dipole‐length electric transition dipole moment, as well as the magnetic transition dipole moment $m=\langle 0\left|\hat{m}\right|j\rangle$ , between the ground state $\left(0\right)$ and an excited state $\left(j\right)$ . The electron magnetic moment operator is $\hat{m}=-\mu_{B}\left(\hat{L}+2\hat{S}\right)$ . The constant $\mu_{B}$ is the Bohr magneton, and $\hat{L}$ and $\hat{S}$ are dimensionless electron orbital and spin angular momentum vector operators, respectively. The magnetic moment operator is therefore in the ‘nonrelativistic with spin’ form and, like the electric dipole moment, not corrected for relativistic picture change. The nonrelativistic with spin form of the magnetic operator is heavily used for theoretical work in molecular magnetism^[62]^ and related fields, indicating that it is a good approximation for valence states. With the corresponding electric transition dipole moment d , the dipole and rotatory strength are [Eq. 1]:

(1) $$D=d\cdot d^{{}^{*}}\;;\;R=Im\left[d\cdot m^{{}^{*}}\right]$$

In cgs‐based Gaussian units, the dipole and rotatory strengths have the same unit of esu^2^cm^2^. Using these units, the dissymmetry factor for the transition is given by:

(2) $$g_{\mathrm{lum}}=4R/D$$

For a parity‐forbidden transition, D should be generalized to include terms from the multipole expansion of the transition probability beyond the electric dipole.^[37]^ For the present study, this was deemed unnecessary.

The radiative decay rate constant k for a transition was calculated according to Equation 2 in the study of Mori et al.,^[10]^ which we reformulate here in terms of the oscillator strength as:

(3) $$k=2\alpha^{3}n^{2}E^{2}f$$

In Equation (3), $f=\left(2/3\right)ED$ is the dimensionless oscillator strength, with E and D being the transition energy and dipole strength in Hartree atomic units, respectively, α is the fine structure constant, and n is the refractive index of the medium. In the calculations, n= 1.42 was chosen, representing the dichloromethane solvent used in the measurements.

The lowest spin triplet states of the examined Pt and Ir complexes exhibit weak zero‐field splitting. The rate constants $k_{i}$ for the individual components of the T_1_ state were Boltzmann averaged, following Mori et al.,^[10]^ at room temperature (298 K). The averaged radiative lifetime τ=1/k was then calculated based on the Boltzmann‐averaged rate constant and converted from atomic units to μs. In a similar fashion, an averaged dissymmetry factor for the emission was computed based on Equation (2) using rotatory and dipole strengths that were Boltzmann‐averaged over the triplet components.

Computational details are provided in a separate section near the end of the article.

## Results and Discussion

### Tris(ethylenediamine)cobalt(III)

**Figure 2:**
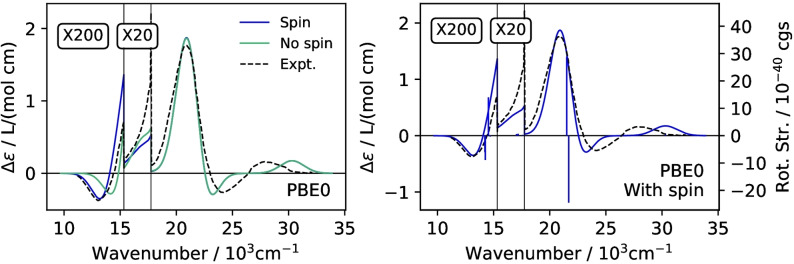
Left: ECD spectra for *lel*_3_‐Λ‐[Co(en)_3_]^3+^ comparing spin‐orbit calculations including (blue, ‘Spin’) or excluding (green, ‘No spin’) the electron spin contributions in the magnetic transition dipole moments. 2c‐TDDFT calculations used the PBE0 functional along with the TDA in the dipole‐length gauge. Right: The ‘stick spectrum’ represents the electronic excitation energies and rotatory strengths. Both panels: Experimental spectrum digitized from Ref. [54]. Δϵ from Gaussian broadening of the transitions with σ=2500 cm^−1^.

See the Computational Section regarding the structure nomenclature *ob* and *lel* and a description of test calculations that were performed for the different conformers with different functionals. We focus here on the results for the *lel*_3_ conformer obtained with 2c‐TDDFT, the Tamm‐Dancoff approximation (TDA), and the PBE0 hybrid functional. The calculated ligand‐field ECD spectrum of *lel*_3_‐Λ‐[Co(en)_3_]^3+^ is displayed in Figure 2 and compared to the experimental spectrum recorded in aqueous solution by Mason and Peart.^[54]^ Additional computed data for Λ‐[Co(en)_3_]^3+^ can be found in the Supporting Information. The *lel*_3_ conformer has *D*_3_ symmetry. The distortion of the coordinating nitrogen atoms from *O_h_* parent symmetry is not very large, such that the spectroscopic properties can be discussed either in terms of the *D*_3_ or the parent *O_h_* symmetry species. Cobalt(III) is a 3d^6^ ion. The complex is low spin, with a filled 3d *t*_2*g*_ sub‐shell. The LF transitions are therefore *t*_2*g*_ to *e_g_*, with excited states of symmetry *T*_1_ and *T*_2_. Distorting the symmetry to *D*_3_ causes the *t*_2*g*_ orbital set to split into *a*_1_ and *e* species. The corresponding *T*_1_ excited LF state splits into *A*_2_ and *E* components, whereas the *T*_2_ excited state splits into *A*_1_ and *E*. The transitions from the *A*_1_ ground state to the *A*_2_ and *E* excited states become electric dipole allowed. The two *E*‐symmetric LF states have been conveniently distinguished as *E_a_* (from *T*_1_) and *E_b_* (from *T*_2_).^[54]^

The un‐magnified portion of the spectra shown in Figure 2 is from the singlet excitations. Based on additional absorption and ECD measurements for a crystal, with different orientation relative to the light polarization/propagation, Mason and Peart^[54]^ assigned the pair of singlet bands between 20 and 25×10^3^ cm^−1^ to the split *T*_1_ state, with *E_a_* (positive *R*) at lower energy and *A*_2_ (negative *R*) at higher energy, and the band close to 30×10^3^ cm^−1^ as *T*_2_/*E_b_*. The first TDDFT calculations of the ECD spectra,^[63]^ in 2003, confirmed the assignment. However, with the BP86 non‐hybrid functional used in the original computation, the LF excitation wavenumbers were too large by several thousand inverse cm. PBE0 gives much better energies for the ligand‐field transitions, for reasons that are discussed in Ref. [57].

In the experimental study, the very weak negative band and the weakly intense shoulder below and above 15×10^3^ cm^−1^ were assigned to the *T*_2_ and *T*_1_ triplet excited states, respectively. Nonrelativistic or scalar relativistic calculations do not give intensity at these energies. As seen in Figure 2, the 2c‐TDDFT TDA PBE0 spectrum including both spin and orbital angular momentum contributions to the magnetic transition dipole moments (blue curve, labeled ‘Spin’) is essentially spot‐on with the experiment, in terms of peak positions as well as in terms of the relative intensities of the triplet versus the singlet transitions. Very importantly, the signs of the rotatory strengths match the signs of the experimental bands. As can be seen in Figure 2, with the chosen broadening, the resulting absolute intensities and peak heights also match the experiment well, both for spin‐allowed and spin‐forbidden transitions.

Calculations were also performed without electron spin contributions in the magnetic moment operator $\hat{m}$ , that is, only using the orbital angular momentum. The latter is the only source of rotatory strength in nonrelativistic and scalar relativistic calculations. It can be seen in Figure 2 that the intensity and band peak positions for the triplet excitations are sensitive to the electron spin contributions in the rotatory strength. Noticeably better agreement between the broadened calculated and the experimental spectrum is obtained when they are included. It is worth mentioning in this context that the peak positions for the triplet as well as the singlet *E_a_* and *A*_2_ pair of bands do not coincide with the excitation energies, as demonstrated by the ‘stick spectrum’ in the right panel of Figure 2. The narrowly split *T*_1_ level with opposite rotatory strengths for *E_a_* and *A*_2_ creates bands with peaks below and above the transition energies, with peak energies or wavenumbers that depend on the rotatory strengths, the transition energy splitting, and the chosen broadening. Differences in the rotatory strengths are responsible for the different positions of the resolved low‐energy ^3^ *E_a_* peak in the calculation without versus with electron spin contributions in the rotatory strength.

### Iridium NHC‐helicene complexes and platinahelicenes

Table 1 summarizes the experimental and calculated emission data for the Ir(III) and Pt(II) systems of Figure 1. The experimentally reported energies correspond to the resolved vibronic peak positions. Compared to the highest experimental values, the 2c‐TDDFT TDA PBE0 vertical transition energies underestimate the experiments consistently by a few tenth of an eV. Given that the decay rate constant *k*, Equation (3), contains a factor *E* ^3^ (one power of *E* is from the oscillator strength), a small underestimation of *E* may translate into a significant underestimation of *k* and thus overestimated phosphorescence lifetime values. This is indeed what the calculations give.

**Table 1:** Experimental and calculated photophysical data for the Ir and Pt systems of Figure 1.^[a]^

	(*P*,Λ_Ir_)‐**A** ^1^	(*P*,Δ_Ir_)‐**A** ^2^	Λ_Ir_‐**A**	*P*‐**3 a**	*P*‐**3 c**
Experimental data					
*E* [eV]	2.36	2.36			
	2.21	2.21	2.49	1.91	1.93
	2.04	2.04			
τ [μs]	350	280	0.53 / 2.4^[b]^	16.5	21
*g*_lum_	3.7×10^−3^	1.5×10^−3^	−9×10^−4^	4.0×10^−3^	3×10^−3^
	(530)	(530)	(493)	( $\lambda_{\max}$ )	(635)^[c]^
Calculated TDDFT TDA PBE0 data					
*E* [eV]^[d]^	2.22	2.22	2.37	1.76	1.79
τ [μs]	452	417	3.9	92.7	69.3
*g*_lum_	8.12×10^−5^	2.07×10^−3^	−1.08×10^−3^	−7.54×10^−4^	−7.20×10^−4^
No spin					
*g*_lum_	1.24×10^−3^	2.81×10^−3^	−1.46×10^−3^	2.21×10^−3^	1.80×10^−3^
With Spin

[a] Measurements at room temperature. Values in parentheses below *g*_lum_ data are the wavelengths in nm at which the dissymmetry factors were measured. Calculations for 298 K using T_1_ equilibrium structures from TDDFT TDA PBE0 geometry optimization (see Tables S11 to S15 for the corresponding xyz coordinates). Calculated *g*_lum_ from Equation (2). Results obtained with the spin‐unrestricted DFT optimized structures are collected in Table S1. Experimental data for (*P*,Λ_Ir_)‐**A** ^1^, (*P*,Δ_Ir_)‐**A** ^2^, and Λ_Ir_‐**A** taken from Ref. [23]. Experimental data for *P*‐**3 a** and *P*‐**3 c** taken from Ref. [30]. Vibronic peak positions provided for the T_1_‐S_0_ emission where resolved. [b] Observed decay kinetics was bi‐exponential at room‐temperature. [c] Measured in dichloromethane solution with a CPL spectrofluorometer constructed in the laboratory at CNRS. The value of +0.013 reported for *P*‐**3 c** in Ref. [30] is likely to be too high. ^[d]^ Vertical T_1_‐S_0_ energies. ZFS was negligible for (*P*,Λ_Ir_)‐**A** ^1^ and (*P*,Δ_Ir_)‐**A** ^2^. The individual triplet component energies (in eV) for the other systems were as follows: Λ_Ir_‐**A** 2.36650, 2.36696, 2.37565; *P*‐**3 a** 1.76391, 1.76409, 1.76445; *P*‐**3 c** 1.79208, 1.79224, 1.79251.

Using full 2c‐TDDFT, that is, without the TDA, led to a stronger underestimation of the transition energies (by an additional 0.2 eV for the platinahelicenes, 0.4 eV for Ir systems **A** ^1/2^, and less than 0.1 eV for Λ_Ir_‐**A**; Table S2 in the Supporting Information). The full 2c‐TDDFT calculations allowed for an assessment of dipole‐length versus dipole‐velocity data, which overall gave reasonable agreement (Table S2). We therefore consider the dipole‐length results from the 2c‐TDDFT TDA calculations to be reliable (see the Computational Section for further details). In the complete basis set limit, the two versions for the electric transition dipole become equivalent, and the length‐gauge rotatory strength becomes origin‐invariant. In practical calculations, that is, with finite basis sets, absent the use of a distributed gauge origin method such as gauge‐including atomic orbitals,^[64]^ the length‐gauge results are origin dependent but typically reliable when the molecules are not far displaced from the origin.^[37]^ The velocity gauge rotatory strength is origin‐invariant by construction, but results may differ considerably from the length gauge for commonly used basis sets. We focus on the dipole‐length data from the TDA calculations in the remainder of the discussion.

A comparison of the results in Table 1 and Table S1 shows that the calculated emission data are not insensitive to the excited‐state structures, but qualitatively the trends between the different sets of calculations and the experiments compare well: At room temperature, τ is several hundred μs for the diastereoisomeric pair of the helicene‐NHC cycloiridiated complexes (*P*,Λ_Ir_)‐**A** ^1^ and (*P*,Δ_Ir_)‐**A** ^2^, only a few μs for its corresponding model NHC system lacking the helicene unit Λ_Ir_‐**A**, and a few ten μs for the Pt‐[8]helicene *P*‐**3 a** and Pt‐[6]helicene *P*‐**3 c**. The calculations give noticeably too large lifetimes for *P*‐**3 a** and *P*‐**3 c**, but correctly identify them to be much smaller than those of (*P*,Λ_Ir_)‐**A** ^1^ and (*P*,Δ_Ir_)‐**A** ^2^. The large increase in the emission lifetime observed for the helicene‐based iridium complexes, compared to the non‐helicenic model, agrees well with the character of their T_1_ excited state, which for the former is strongly delocalized across the π‐helical ligand and demonstrates the reduced contribution of the metal orbitals in the emission transition.^[23]^

The calculated $g_{\mathrm{lum}}$ (including electron spin contributions) are within a factor of two from the experimental estimates [within a factor of 3 for (*P*,Λ_Ir_)‐**A** ^1^], which can be considered satisfactory. The electron spin contributions in the magnetic transition dipole are seen to be very important for (*P*,Λ_Ir_)‐**A** ^1^, raising $g_{\mathrm{lum}}$ by more than a factor of 10. For the platinahelicenes, the electron spin contributions are critical, because without them the rotatory strengths do not have the correct signs. Reference [30] previously reported $g_{\mathrm{lum}}=$ +0.013, much larger than the computed value. However, a revised experimental $g_{\mathrm{lum}}$ of +0.003 is close to what we calculate.

It is important to note that for the iridium complexes, the computations (both with and without the electron spin contributions in the rotatory strength) reproduce correctly the trend in sign of measured $g_{\mathrm{lum}}$ for (*P*,Λ_Ir_)‐**A** ^1^ and (*P*,Δ_Ir_)‐**A** ^2^ (positive) versus Λ_Ir_‐**A** (negative). This supports the finding drawn from the experimental data that the sign of the CPL signal of **A** ^1,2^ is controlled by the stereochemistry of the π‐helical NHC ligand, rather than stereochemistry at the metal.^[23]^ For the platinahelicenes, the sign of $g_{\mathrm{lum}}$ also follows the stereochemistry of the helicene unit (*P* – positive, *M* – negative). However, the Pt center, because it is part of the helicene, participates more directly in the helical π‐chromophore.^[30]^ This is likely one of the main reasons why the sign of $g_{\mathrm{lum}}$ is not correctly predicted by the calculations that do not incorporate the electron spin in the magnetic transition dipole moment.

## Conclusion

The calculation of magnetic transition dipole moments, rotatory strengths, and velocity‐gauge electric transition dipole moments was implemented in the two‐component relativistic TDDFT module of the ADF package. The functionality will be available in the next release of ADF. Relativistic effects are treated in the computations via the all‐electron ZORA quasirelativistic Hamiltonian, and they enter the associated frequency‐dependent linear response to determine excitation energies and transition moments. The circular dichroism of the spin‐forbidden and spin‐allowed ligand‐field transitions of tris(ethylenediamine)cobalt(III) modeled in this way agreed very well with measurements by Mason and Peart.^[54]^ Phosphorescence dissymmetry factors $g_{\mathrm{lum}}$ and the corresponding lifetimes were calculated for three iridium complexes bearing helicenic or non‐helicenic N‐heterocyclic‐carbene ligand and for two platinahelicenes. The agreement with experimental data was overall satisfactory, showing promise for future applications of the methodology to similar systems.[^65^, ^66^] The calculations reproduced the signs and order of magnitude of $g_{\mathrm{lum}}$ , and the large variations of phosphorescence lifetimes among the complexes. The role of the electron spin contributions to the magnetic transition dipole moment was shown to be very important for one of the Ir systems and both platinahelicenes. For the latter, $g_{\mathrm{lum}}$ had the wrong sign when the electron spin contributions were not considered. Even for the cobalt complex, which exhibits comparatively smaller relativistic effects because of the lower nuclear charge of the metal, the electron spin contributions improved the appearance of the spin‐forbidden ECD in comparison with the experimental spectrum. Further improvements of the calculations will likely be obtained by treating vibronic effects in the spectra. Work along these lines is being pursued in our laboratory.

## Computational Section

Triplet excited‐state structures for the Ir and Pt complexes of Figure 1 were optimized with scalar ZORA spin‐unrestricted DFT. Calculations used the PBE0^[67]^ hybrid functional (25 % global exact exchange) along with the TZ2P basis for the metal, DZ for hydrogens, and DZP otherwise, for optimizations and TDDFT runs, and employed a continuum solvent model with the dielectric constant for dichloromethane.^[68]^ TDDFT response computations were based on the S_0_ closed‐shell ground state at the T_1_ equilibrium structure, using spin‐orbit ZORA and the 2c‐TDDFT module with the ‘FullKernel’ option to determine the vertical transition energies and transition moments needed in Equations (1)–(3). The TDDFT calculations employed the Tamm‐Dancoff approximation (TDA).^[69]^ In agreement with observations made previously by Peach et al.,^[70]^ and confirmed by us for some helicene‐based metal complexes,[^30^, ^71^, ^72^] computations not employing the TDA underestimated the T_1_ energies significantly. The calculated total energy differences between the S_0_ and T_1_ states at the spin‐unrestricted DFT‐optimized T_1_ equilibrium structures likewise underestimated the emission energies substantially. Accordingly, an alternate set of T_1_ equilibrium structures was determined by TDDFT TDA PBE0 optimizations, using the Gaussian 09 program, revision D.03,^[73]^ and def2‐SV(P) Gaussian‐type basis sets^[74]^ with matching scalar relativistic effective core potentials for Ir and Pt. These structures were used for the 2c‐TDDFT calculations reported herein. For completeness, results obtained with the spin‐unrestricted DFT optimized T_1_ structures are provided in the Supporting Information, Table S1. In the present implementation, velocity gauge rotatory strengths are not available in conjunction with the TDA. Extensive test calculations with full TDDFT, in one‐ or two‐component form, as documented in Section S2 in the Supporting Information for the cobalt complex, showed good agreement between the length and velocity gauges for center‐of‐nuclear‐charge (CNC) coordinates, and demonstrate the desired origin‐invariance of the velocity‐gauge results. To minimize errors from the origin dependence of the dipole‐length gauge rotatory strengths in the TDA calculations, molecules were therefore placed at the CNC. Test calculations for *P*‐**3 c** with the platinum atom versus CNC at the coordinate origin (Tables S3 and S4 in the Supporting Information) showed that, while there is an origin dependence, it does not qualitatively affect the conclusions.

In reference to an actual or approximate (depending on the conformer) 3‐fold rotational axis of symmetry of tris(ethylenediamine)cobalt(III), the C−C backbone of the ligand can either be approximately parallel (*lel*) or oblique (*ob*), giving rise to 4 conformers for each of the Δ and Λ configurations: *lel*_3_, *lel*_2_ *ob*_1_, *lel*_1_ *ob*_2_, and *ob*_3_. The conformers structures of the complex [Co(en)_3_]^3+^ were optimized with three different functionals, namely BP86,[^75^, ^76^] B3LYP,[^77^, ^78^] and PBE0, to examine the sensitivity of the calculated spectrum to the underlying structural parameters. The [Co(en)_3_]^3+^ spectra were simulated using the same set of functionals, with and without the TDA. In all these computations the aforementioned basis set combination (TZ2P/DZP/DZ) was used and a continuum solvent model (COSMO) was employed to simulate solvent (water) effects. With the B3LYP functional, the different conformers were within less than 1 kcal/mol in energy and therefore the calculations do not reliably identify a most abundant conformer, in agreement with NMR studies.[^79^, ^80^] In agreement with Ref. [63], solvent effects on the ligand‐field transition were weak. Moreover, the ligand‐field ECD spectra for the different conformers were very similar, and the underlying dependence of the spectra on the functional used for the structure optimizations was weak. In agreement with Ref. [57], the PBE0 functional performed best for the spectrum. TDA calculations showed a marked improvement of the triplet excitations. For all these reasons, most of the data computed for Λ‐[Co(en)_3_]^3+^ are collected in the Supporting Information, and we focus the discussion on the gas‐phase TDA PBE0 spectrum of the B3LYP‐optimized *lel*_3_ conformer. Note that *lel*_3_ is the conformer adopted in crystals containing the complex on which ECD measurements were carried out previously.^[54]^

## Conflict of interest

The authors declare no conflict of interest.

1

## Supporting information

As a service to our authors and readers, this journal provides supporting information supplied by the authors. Such materials are peer reviewed and may be re‐organized for online delivery, but are not copy‐edited or typeset. Technical support issues arising from supporting information (other than missing files) should be addressed to the authors.

Supporting Information

## Data Availability

The data that support the findings of this study are available from the corresponding author upon reasonable request.
