# Supplementary material for: Optical Activity of Spin‐Forbidden Electronic Transitions in Metal Complexes from Time‐Dependent Density Functional Theory with Spin‐Orbit Coupling
Source: ChemistryOpen. 2022 May 18;11(5):e202200020. doi: 10.1002/open.202200020 (PMC9117156; doi:10.1002/open.202200020)
Supplement: Supplementary file 1 — Supporting Information [file OPEN-11-e202200020-s001.pdf]

ChemistryOpen

Supporting Information

Optical Activity of Spin-Forbidden Electronic Transitions in Metal Complexes from Time-Dependent Density Functional Theory with Spin-Orbit Coupling

Herbert D. Ludowieg, Monika Srebro-Hooper, Jeanne Crassous, and Jochen Autschbach*

S1 Additional Ir and Pt complex data

Table S1: Experimental and calculated photophysical data for the systems of Figure 1. SP-TDDFT/TDA PBE0 calculations based on spin-unrestricted DFT-optimized T_1 equilibrium geometries.[a]

	(P,Λ_{Ir})-**A**[1]	(P,Δ_{Ir})-**A**[2]	Λ_{Ir}-**A**	P-**3a**	P-**3c**
Experimental data					
E / eV	2.3616	2.35711			
	2.20612	2.20612	2.48964	1.91334	1.92522
	2.04257	2.04257			
τ / μs	350	280	0.53 / 2.4[b]	16.5	21
$g_{lum,av}$	3.7×10^{-3}	1.5×10^{-3}	-9×10^{-4}	4.0×10^{-3}	3×10^{-3}
	at 530 nm	at 530 nm	at 493 nm	at λ_{max}	at 635 nm[c]
Calcd. TDA-TDDFT/PBE0 at Spin Unrestricted PBE0-optimized T_1[d]					
E / eV[e]	2.11	2.11	2.36	1.68	1.72
τ / μs length	648	607	3.7	125	84.7
$g_{lum,av}$ length / No spin	4.64×10^{-5}	1.90×10^{-3}	-8.36×10^{-4}	-1.47×10^{-3}	-6.45×10^{-4}
$g_{lum,av}$ length / Spin	1.54×10^{-3}	2.90×10^{-3}	-1.08×10^{-3}	1.05×10^{-3}	1.30×10^{-3}

[a] Room temperature. Data for (P,Λ_{Ir})-**A**[1], (P,Δ_{Ir})-**A**[2], and Λ_{Ir}-**A** taken from Reference 23. Data for P-**3a** and P-**3c** taken from Reference 30. Vibronic peak positions for the T_1-S_0 emission where resolved. [b] Observed decay kinetics was bi-exponential at room-temperature. [c] Measured in dichloromethane solution with a CPL spectrofluorometer constructed in the laboratory at CNRS. The value of +0.013 reported for P-**3c** in Reference 30 is therefore likely to be too high. [d] See Tables S16 to S20 for xyz structures. [e] Vertical T_1-S_0 energies. ZFS was negligible for (P,Λ_{Ir})-**A**[1] and (P,Δ_{Ir})-**A**[2]. The individual triplet component energies (in eV) for the other systems were as follows: Λ_{Ir}-**A** 2.35934, 2.35987, 2.36926; P-**3a** 1.68337, 1.68355, 1.68383; P-**3c** 1.72028, 1.72043, 1.72066.

Table S2: Experimental and calculated photophysical data for the systems of Figure 1. 2c-TDDFT calculations performed at the PBE0 level without TDA.[a]

	(P,Λ_{Ir})-**A**[1]	(P,Δ_{Ir})-**A**[2]	Λ_{Ir}-**A**	P-**3a**	P-**3c**
Experimental data					
E / eV	2.3616	2.35711			
	2.20612	2.20612	2.48964	1.91334	1.92522
	2.04257	2.04257			
τ / μs	350	280	0.53 / 2.4[b]	16.5	21
$g_{lum,av}$	3.7×10^{-3}	1.5×10^{-3}	-9×10^{-4}	4.0×10^{-3}	3×10^{-3}
	at 530 nm	at 530 nm	at 493 nm	at $\lambda_{\max}$	at 635 nm[c]
Calcd. TDDFT/PBE0 at TDA-TDDFT/PBE0-optimized T_1[d]					
E / eV[e]	1.86	1.86	2.31	1.56	1.59
τ / μs length	4081	3647	3.13	212	150
τ / μs velocity	3397	3224	3.11	227	162
$g_{lum,av}$ length / no-spin	5.73×10^{-4}	3.94×10^{-3}	-5.55×10^{-4}	-1.79×10^{-3}	-1.53×10^{-3}
$g_{lum,av}$ length / spin	4.34×10^{-3}	6.20×10^{-3}	-7.32×10^{-4}	1.91×10^{-3}	1.47×10^{-3}
$g_{lum,av}$ velocity / spin	3.30×10^{-3}	6.01×10^{-3}	-7.34×10^{-4}	2.27×10^{-3}	1.96×10^{-3}

[a] Room temperature. Data for (P,Λ_{Ir})-**A**[1], (P,Δ_{Ir})-**A**[2], and Λ_{Ir}-**A** taken from Reference 23. Data for P-**3a** and P-**3c** taken from Reference 30. Vibronic peak positions for the T_1-S_0 emission where resolved. [b] Observed decay kinetics was bi-exponential at room-temperature. [c] Measured in dichloromethane solution with a CPL spectrofluorometer constructed in the laboratory at CNRS. The value of +0.013 reported for P-**3c** in Reference 30 is therefore likely to be too high. [d] See Tables S16 to S20 for xyz structures. [e] Vertical T_1-S_0 energies. ZFS was negligible for (P,Λ_{Ir})-**A**[1] and (P,Δ_{Ir})-**A**[2]. The individual triplet component energies (in eV) for the other systems were as follows: Λ_{Ir}-**A** 2.30494, 2.30536, 2.31477; P-**3a** 1.56385, 1.56402, 1.56426; P-**3c** 1.59232, 1.59246, 1.59266.

Table S3: Comparison of the SO-TDDFT/TDA rotatory strengths for *P*-**3c** calculated with the coordinate origin at the center of nuclear charges (R_{CNC}) or at Pt (R_{Pt}). The metal center is at the xyz coordinates of (1.88557 Å, -0.18819 Å, -0.09184 Å) relative to the CNC. The notation EXX means $\times 10^{XX}$.

E / eV	R_{CNC} / cgs	R_{Pt} / cgs
1.79208	-1.75E-02	-1.78E-02
1.79224	7.71E-03	11.9E-03
1.79251	11.3E-02	9.94E-02

Table S4: Comparison of the SO-TDDFT/TDA $g_{lum,av}$ values for *P*-**3c** using the rotatory strengths calculated with the coordinate origin coinciding with the CNC or the Pt atom. The metal center is at the xyz coordinates of (1.88557 Å, -0.18819 Å, -0.09184 Å) relative to the CNC.

Origin	$g_{lum,av}$
CNC	1.80E-03
Pt	1.63E-03

S2 Additional Λ-$[Co(en)_3]^{3+}$ data

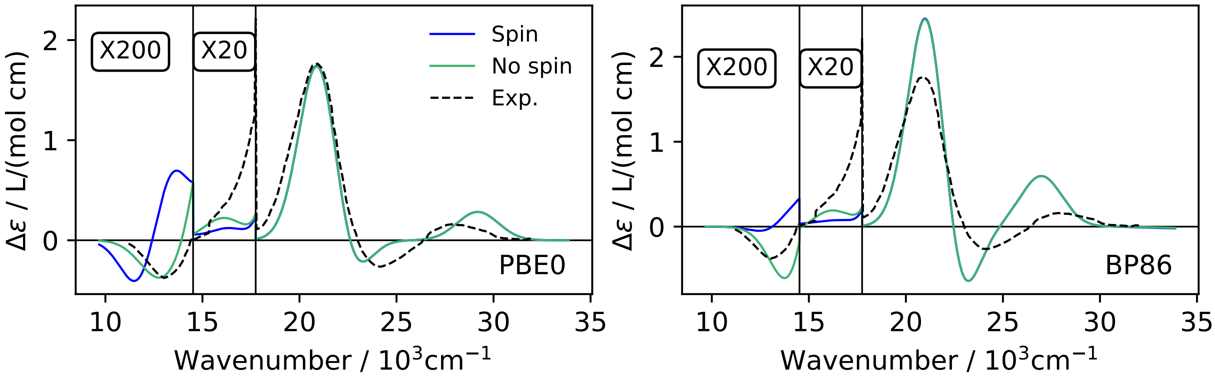

Figure S1: ECD spectra for *lel*$_3$ Λ-$[Co(en)_3]^{3+}$ comparing spin-orbit calculations with the inclusion of the spin operator in the magnetic dipole (blue line) and without (green line) for PBE0 (left) and BP86 (right) functionals for full 2c-TDDFT calculations, i.e., not using the TDA. Calculations were performed in the dipole-length gauge. Both figures used a structure optimized with the B3LYP DFT functional. Experimental values taken from Reference 54. Calculated energies from PBE0 were shifted by 161 cm^{-1} and those from BP86 were shifted by –2420 cm^{-1}. Broadening of the spectra was simulated with Gaussian functions with a value of σ = 2500 cm^{-1}.

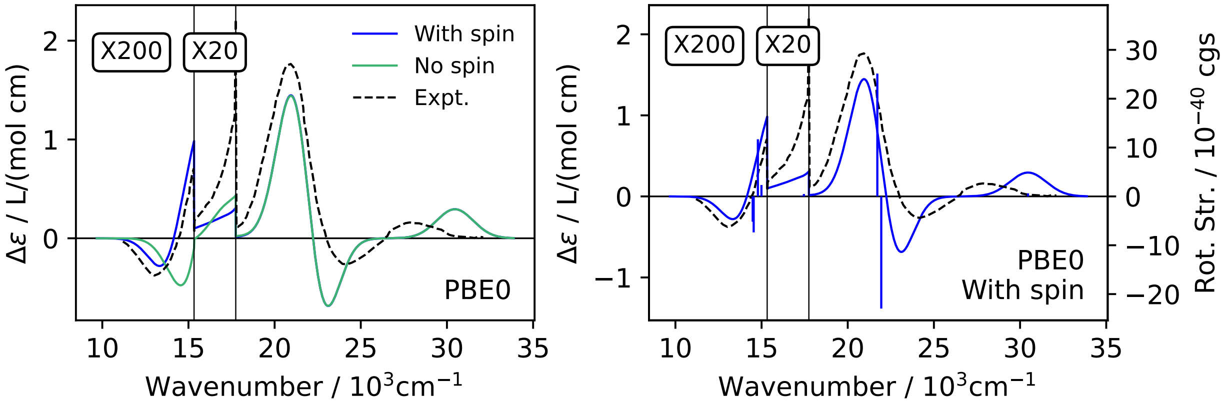

Figure S2: ECD spectra for *lel*$_3$ Λ-$[Co(en)_3]^{3+}$ comparing spin-orbit calculations including (blue) or excluding (green) the spin operator in the magnetic dipole. 2c-TDDFT calculations used the PBE0 functional along with the TDA in the dipole-length gauge in a water solvent simulated with a COSMO. Stick spectra in the right panel are the rotatory strengths in cgs scaled by the respective scaling factor. Experimental values taken from Reference 54. Broadening of the spectra was simulated with Gaussian functions with a value of σ = 2500 cm^{-1}.

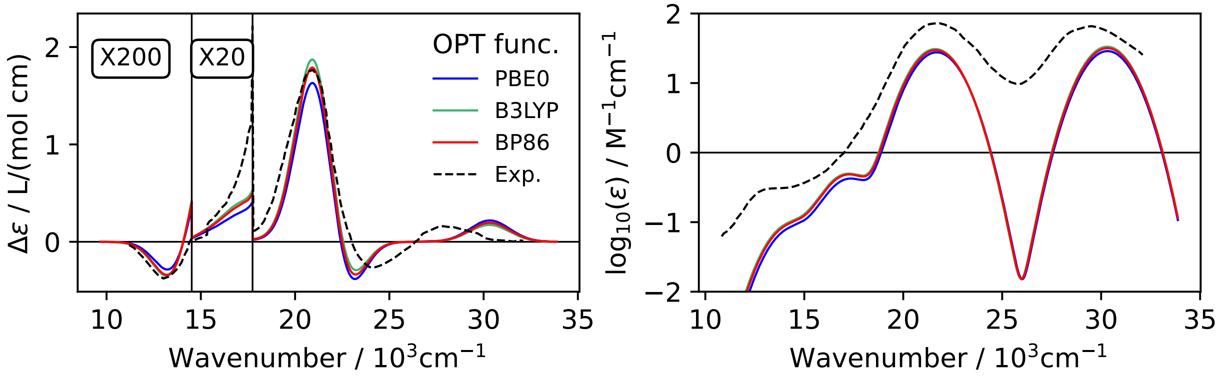

Figure S3: ECD (left) and absorption (right) spectra for *lel*$_3$ Λ-[Co(en)$_3$]$^{3+}$ using the PBE0 functional in the 2c-TDDFT calculation and structures optimized with the respective functionals. All 2c-TDDFT calculations employed the TDA in the dipole-length gauge. Experimental values taken from Reference 54. Vertical excitation energies for the blue and red lines were red-shifted by 1410 cm^{-1} and 280 cm^{-1}, respectively. The energies for the absorption spectra were shifted by the same energy value. Broadening of the spectra was simulated with Gaussian functions with a value of σ = 2500 cm^{-1}.

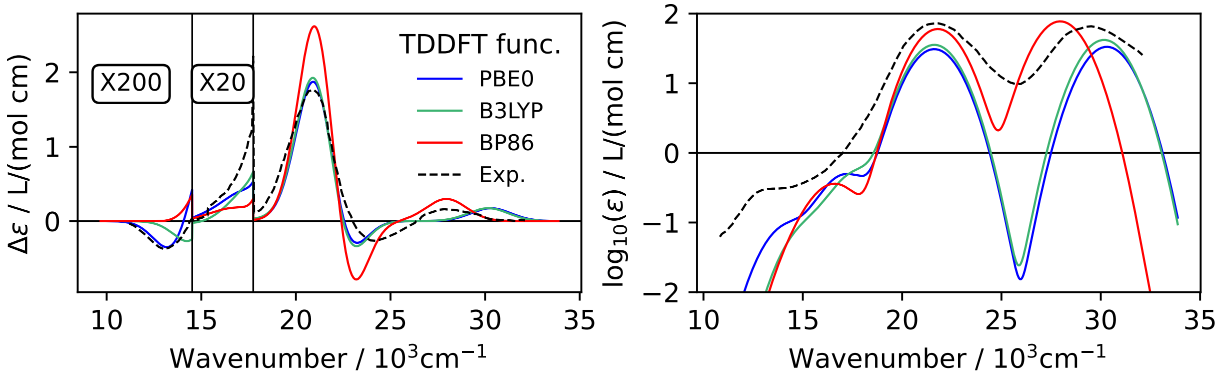

Figure S4: ECD (left) and absorption (right) spectra for *lel*$_3$ Λ-[Co(en)$_3$]$^{3+}$ using a different DFT functional in the 2c-TDDFT computation using a structure optimized at the B3LYP level of theory. Calculations performed in the dipole-length gauge. Experimental values taken from Reference 54. Vertical excitation energies calculated with the B3LYP and BP86 functionals were red-shifted by 950 cm^{-1} and 2420 cm^{-1}, respectively. The energies for the absorption spectra were shifted by the same energy value. Broadening of the spectra was simulated with Gaussian functions with a value of σ = 2500 cm^{-1}.

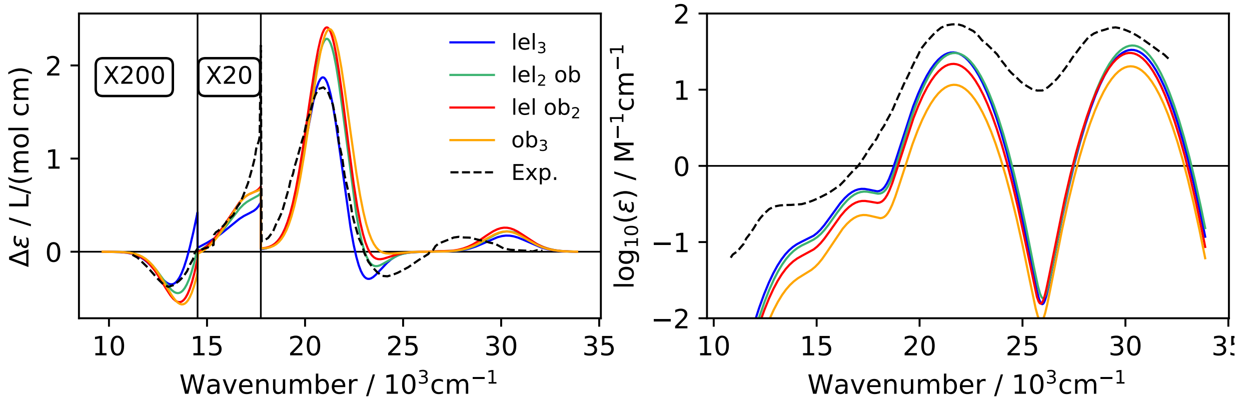

Figure S5: ECD (left) and absorption (right) spectra for all four conformers of Λ-$[Co(en)_3]^{3+}$. Each structure was optimized at the B3LYP level of theory and 2c-TDDFT calculations used the PBE0 functional along with the TDA in the dipole-length gauge. Experimental values taken from Reference 54. Broadening of the spectra was simulated with Gaussian functions with a value of $\sigma = 2500$ cm^{-1}.

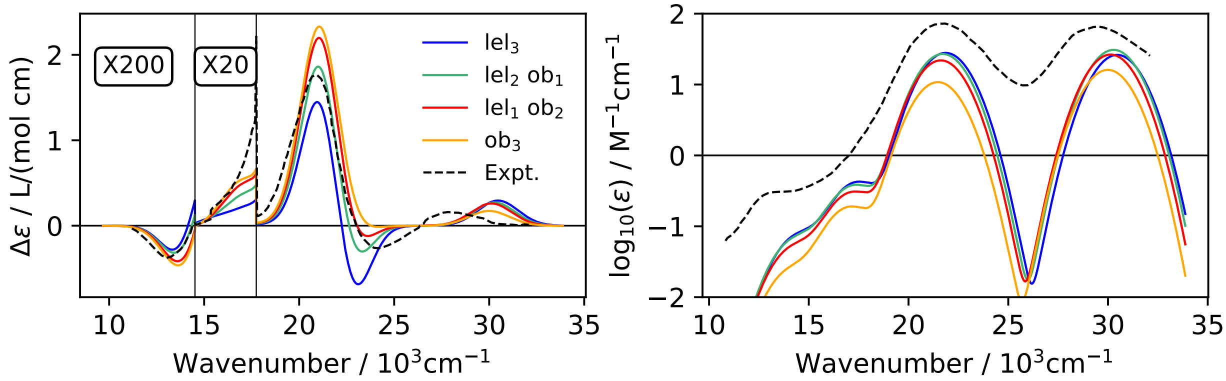

Figure S6: ECD (left) and absorption (right) spectra for all four conformers of Λ-$[Co(en)_3]^{3+}$. Each structure was optimized at the B3LYP level of theory and 2c-TDDFT calculations used the PBE0 functional along with the TDA in a water solvent simulated with a COSMO. Calculations were performed in the dipole-length gauge. Calculated energies were red-shifted by 1570 cm^{-1}, 1690 cm^{-1}, 1770 cm^{-1}, and 1940 cm^{-1} for the blue, green and red lines, respectively. Experimental values taken from Reference 54. Broadening of the spectra was simulated with Gaussian functions with a value of $\sigma = 2500$ cm^{-1}.

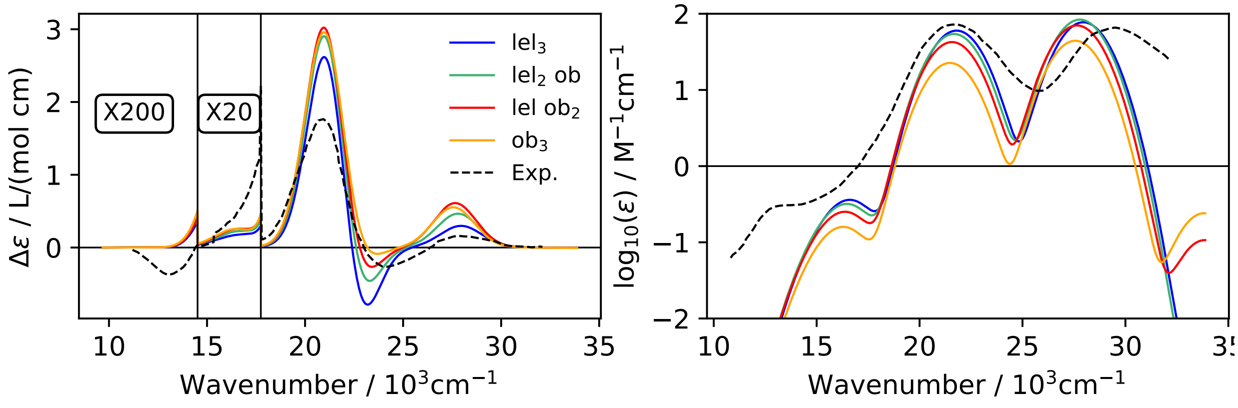

Figure S7: ECD (left) and absorption (right) spectra for all four conformers of Λ-$[Co(en)_3]^{3+}$. Each structure was optimized at the B3LYP level of theory and 2c-TDDFT calculations used the BP86 functional along with the TDA in the dipole-length gauge. Experimental values taken from Reference 54. Calculated energies for the blue, green, red, and orange lines were red-shifted by 2420 cm^{-1}, 2540 cm^{-1}, 2640 cm^{-1}, and 2780 cm^{-1}, respectively. The energies for the absorption spectra were shifted by the same energy value. Broadening of the spectra was simulated with Gaussian functions with a value of $\sigma = 2500$ cm^{-1}.

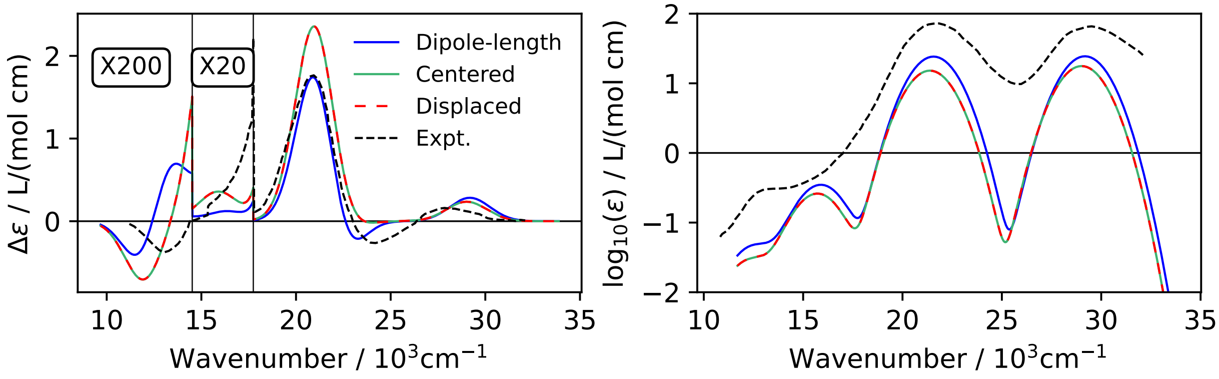

Figure S8: ECD (left) and absorption (right) spectra of *lel*$_3$ Λ-$[Co(en)_3]^{3+}$ using the PBE0 functional in the 2c-TDDFT calculation and a structure optimized with the B3LYP functional. Experimental values taken from Reference 54. Blue line represents the calculation in the dipole-length gauge. Both the green and red lines are in the velocity gauge with the metal centered at the coordinate origin (0, 0, 0) or displaced at (1.43 Å, -5.08 Å, 2.22 Å). Calculated energies were not shifted. Broadening of the spectra was simulated with Gaussian functions with a value of $\sigma = 2500$ cm^{-1}.

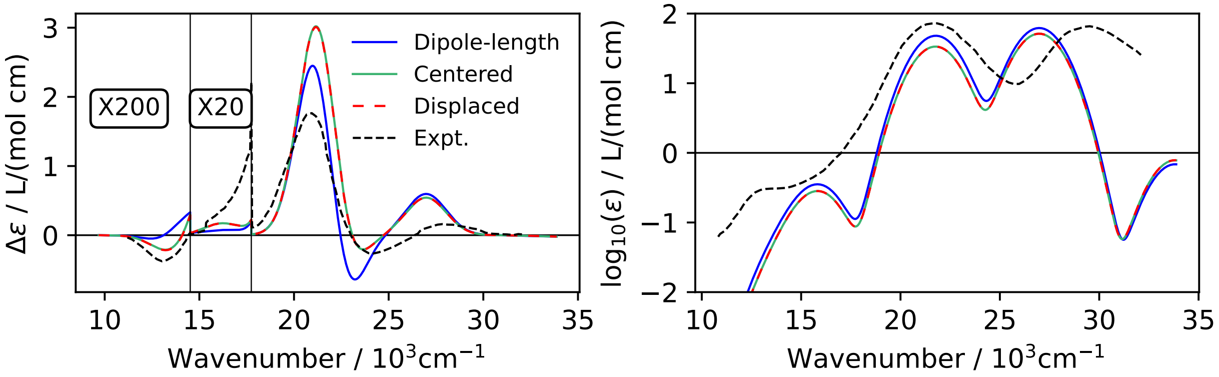

Figure S9: ECD (left) and absorption (right) spectra of *lel*$_3$ Λ-$[Co(en)_3]^{3+}$ using the BP86 functional in the 2c-TDDFT calculation and a structure optimized with the B3LYP functional. Experimental values taken from Reference 54. Blue line represents the calculation in the dipole-length gauge. Both the green and red lines are in the velocity gauge with the metal centered at the coordinate origin (0, 0, 0) or displaced at (1.43 Å, -5.08 Å, 2.22 Å). Calculated energies were red-shifted by 2420 cm^{-1}. Broadening of the spectra was simulated with Gaussian functions with a value of $\sigma = 2500\ cm^{-1}$.

Table S5: Energies and rotatory strengths of Λ-$[Co(en)_3]^{3+}$ calculated with the PBE0 functional in a water solvent with the TDA. 'cgs' stands for units of 10^{-40}esu^{2}cm^2. The 'Spin' and 'No spin' are for calculations which either include or exclude the spin operator in the formalism, respectively. Data for Figure S2.

		Rotatory Strength	
Excitation	Energy / cm^{-1}	No spin / cgs	Spin / cgs
1	14450	-1.3054×10^{-3}	-2.5905×10^{-2}
2	14500	9.2680×10^{-3}	-3.6820×10^{-2}
3	14560	4.9413×10^{-5}	-1.0141×10^{-5}
4	14750	-1.8296×10^{-2}	5.8493×10^{-2}
5	14950	-3.2201×10^{-3}	1.1639×10^{-2}
6	15140	-1.2565×10^{-7}	-1.2921×10^{-5}
7	17260	4.2637×10^{-3}	4.7517×10^{-3}
8	17410	0.10584	0.10480
9	17420	-4.9748×10^{-2}	-8.0003×10^{-2}
10	17730	-2.5327×10^{-5}	-2.7659×10^{-5}
11	17820	1.3005×10^{-2}	1.6425×10^{-2}
12	17960	-4.0399×10^{-3}	-3.3378×10^{-3}
13	21680	25.073	25.149
14	21900	−22.900	−22.964
15	30420	0.62390	0.62410
16	30520	-2.3909×10^{-4}	-2.3796×10^{-4}

Table S6: Energies and rotatory strengths of Λ-$[Co(en)_3]^{3+}$ calculated with the PBE0 functional in the gas phase with the TDA. 'cgs' stands for units of 10^{-40}esu^{2}cm^2. The 'Spin' and 'No spin' are for calculations which either include or exclude the spin operator in the formalism, respectively. Data for Figure 2.

		Rotatory Strength	
Excitation	Energy / cm^{-1}	No spin / cgs	Spin / cgs
1	12610	3.6679×10^{-4}	-3.0308×10^{-2}
2	12660	8.7720×10^{-3}	-3.5311×10^{-2}
3	12720	4.8928×10^{-5}	1.2747×10^{-4}
4	12930	-1.9242×10^{-2}	7.0106×10^{-2}
5	13080	3.9912×10^{-4}	-1.8113×10^{-3}
6	13300	-7.3675×10^{-7}	-1.5661×10^{-5}
7	15430	2.5775×10^{-2}	2.9040×10^{-2}
8	15560	−0.18036	−0.21308
9	15570	0.24545	0.24854
10	15900	-3.0768×10^{-5}	-3.3316×10^{-5}
11	15980	1.0286×10^{-2}	1.3128×10^{-2}
12	16140	-2.3518×10^{-3}	-1.9415×10^{-3}
13	19900	28.768	28.861
14	20080	−24.431	−24.505
15	28690	0.37020	0.37017
16	28780	-5.2843×10^{-4}	-5.3114×10^{-4}

Table S7: Energies, rotatory strengths, and oscillator strengths of *lel*$_3$ Λ-$[Co(en)_3]^{3+}$ calculated with the PBE0 functional in the gas phase with TDA. Calculations were performed that the relativistic scalar and spin-orbit level with the ZORA Hamiltonian. 'cgs' stands for units of 10^{-40}esu^{2}cm^2. All values were calculated in the velocity gauge.

		Rotatory Strength / cgs		Oscillator Strength	
E_{Scalar} / cm^{-1}	E_{SO} / cm^{-1}	Scalar	Spin-Orbit	Scalar	Spin-Orbit
19820	19870	0.51504	0.64127	8.4944×10^{-8}	1.3326×10^{-7}
19830	19920	0.53031	0.87274	9.0148×10^{-8}	1.9898×10^{-6}
20000	20080	18.710	18.072	1.1601×10^{-4}	1.1156×10^{-4}
28660	28680	0.15932	0.14758	1.4993×10^{-4}	1.4988×10^{-4}
28670	28700	0.15950	0.18039	1.5002×10^{-4}	1.5105×10^{-4}

Table S8: Energies, rotatory strengths, and oscillator strengths of *lel*$_3$ Λ-$[Co(en)_3]^{3+}$ calculated with the PBE0 functional in the gas phase with TDA. Calculations were performed that the relativistic scalar and spin-orbit level with the ZORA Hamiltonian. 'cgs' stands for units of 10^{-40}esu^{2}cm^2. All values were calculated in the length gauge.

		Rotatory Strength / cgs		Oscillator Strength	
E_{Scalar} / cm^{-1}	E_{SO} / cm^{-1}	Scalar	Spin-Orbit	Scalar	Spin-Orbit
19820	19870	14.899	14.762	7.1083×10^{-5}	7.0616×10^{-5}
19830	19920	14.909	14.099	7.1183×10^{-5}	7.4117×10^{-5}
20000	20080	−25.416	−24.505	2.1407×10^{-4}	2.1137×10^{-4}
28660	28680	0.17985	0.16636	1.9108×10^{-4}	1.9043×10^{-4}
28670	28700	0.17998	0.20370	1.9103×10^{-4}	1.9261×10^{-4}

Table S9: Energies, rotatory strengths, and oscillator strengths of *lel*$_3$ Λ-$[Co(en)_3]^{3+}$ calculated with the BP86 functional in the gas phase with TDA. Calculations were performed that the relativistic scalar and spin-orbit level with the ZORA Hamiltonian. 'cgs' stands for units of 10^{-40}esu^{2}cm^2. All values were calculated in the velocity gauge.

		Rotatory Strength / cgs		Oscillator Strength	
E_{Scalar} / cm^{-1}	E_{SO} / cm^{-1}	Scalar	Spin-Orbit	Scalar	Spin-Orbit
22390	22420	−5.8444	−5.7313	1.6145×10^{-5}	1.5577×10^{-5}
22390	22460	−5.8560	−5.5574	1.6211×10^{-5}	1.8873×10^{-5}
22660	22710	29.742	29.322	4.4041×10^{-4}	4.3421×10^{-4}
28730	28750	0.15497	0.1447	9.1767×10^{-5}	9.2124×10^{-5}
28730	28770	0.15498	0.1702	9.1804×10^{-5}	9.2521×10^{-5}

Table S10: Energies, rotatory strengths, and oscillator strengths of *lel*$_3$ Λ-$[Co(en)_3]^{3+}$ calculated with the BP86 functional in the gas phase with TDA. Calculations were performed that the relativistic scalar and spin-orbit level with the ZORA Hamiltonian. 'cgs' stands for units of 10^{-40}esu^{2}cm^2. All values were calculated in the length gauge.

		Rotatory Strength / cgs		Oscillator Strength	
E_{Scalar} / cm^{-1}	E_{SO} / cm^{-1}	Scalar	Spin-Orbit	Scalar	Spin-Orbit
22390	22420	17.034	16.982	1.3715×10^{-4}	1.3676×10^{-4}
22390	22460	17.044	16.752	1.3731×10^{-4}	1.4062×10^{-4}
22660	22710	−29.012	−28.651	4.1905×10^{-4}	4.1795×10^{-4}
28730	28750	0.33995	0.31694	4.4161×10^{-4}	4.4150×10^{-4}
28730	28770	0.33987	0.37433	4.4150×10^{-4}	4.4760×10^{-4}

S3 XYZ coordinates

S3.1 Pt and Ir complexes TDDFT/TDA optimized T_1 geometries

Table S11: XYZ coordinates for *P*-**3a**

Symbol	X	Y	Z
C	-2.03058	1.39646	-0.37363
C	-0.82010	0.66174	-0.70487
C	0.49209	1.26627	-0.44992
C	0.57117	2.63327	-0.32089
C	-0.60677	3.42143	-0.40609
C	-1.90699	2.81860	-0.40499
C	-3.29565	0.81369	-0.07028
C	-4.46899	1.62982	-0.23661
C	-4.32142	3.02857	-0.38485
C	-3.07799	3.61801	-0.40456
C	-3.49581	-0.57056	0.35294
C	-4.75130	-1.16391	0.04334
C	-5.87148	-0.34433	-0.29700
C	-5.74865	1.01774	-0.33143
C	-2.51271	-1.38140	1.02706
C	-2.63543	-2.79379	0.93634
C	-3.83124	-3.37154	0.42881
C	-4.89138	-2.57846	0.08339
C	-1.43714	-0.86003	1.85888
C	-0.38324	-1.73072	2.26888
C	-0.44918	-3.12265	1.95268
C	-1.56702	-3.63963	1.36922
C	-1.44294	0.45060	2.39831
C	-0.42176	0.90584	3.20888
C	0.67014	0.07252	3.51847
C	0.67731	-1.22829	3.06119
C	-0.74230	-0.57919	-1.39127
N	0.52244	-1.14352	-1.37523
C	0.74343	-2.32268	-1.96028
C	-0.25415	-3.01477	-2.63753
C	-1.53141	-2.43370	-2.73536
C	-1.77443	-1.22618	-2.11296
Pt	1.94216	-0.03935	-0.50865
O	3.29306	1.20447	0.34596
C	4.51703	0.93569	0.57947

Continued on next page

Table S11 – *Continued from previous page*

Symbol	X	Y	Z
C	5.18133	-0.26777	0.29831
C	4.62637	-1.41607	-0.30191
C	5.50447	-2.61661	-0.53356
O	-0.60069	4.76356	-0.41073
C	0.62084	5.46154	-0.37450
O	3.42421	-1.54281	-0.67895
C	5.27847	2.06227	1.22314
H	1.76572	-2.70253	-1.87891
H	-0.02403	-3.97635	-3.10094
H	-2.32373	-2.92834	-3.30411
H	-2.74893	-0.74251	-2.18848
H	1.54480	3.10244	-0.16488
H	-2.97556	4.70234	-0.47773
H	-5.22194	3.64334	-0.48255
H	-6.62249	1.65561	-0.49709
H	-6.83744	-0.82677	-0.47321
H	-5.85046	-3.01971	-0.20486
H	-3.91078	-4.46211	0.38347
H	-1.66687	-4.71840	1.21343
H	0.37694	-3.77272	2.25655
H	1.49048	-1.90800	3.33505
H	1.48820	0.44467	4.14238
H	-0.46850	1.91997	3.61696
H	-2.28744	1.10972	2.19441
H	4.78822	2.33867	2.17305
H	6.32935	1.80470	1.42006
H	5.23990	2.95111	0.56911
H	6.23729	-0.31667	0.56988
H	5.06532	-3.49303	-0.02551
H	5.52747	-2.84781	-1.61321
H	6.53368	-2.46827	-0.17496
H	0.36609	6.52960	-0.43352
H	1.16650	5.27121	0.56711
H	1.26394	5.19161	-1.23127

Table S12: XYZ coordinates for *P*-**3c**

Symbol	X	Y	Z
Pt	1.88557	-0.18819	-0.09184

Continued on next page

Table S12 – *Continued from previous page*

Symbol	X	Y	Z
N	0.53248	-1.39044	-0.93333
O	-0.83139	4.50753	-0.36816
O	3.16072	1.14825	0.73682
O	3.42989	-1.63253	-0.16462
C	0.38349	1.05819	-0.12840
C	0.40946	2.43348	-0.07897
H	1.35956	2.94635	0.08566
C	-0.78729	3.16948	-0.27172
C	-2.06683	2.51788	-0.28913
C	-3.26016	3.26301	-0.43357
H	-3.19230	4.33714	-0.61567
C	-4.48585	2.63006	-0.39893
H	-5.40156	3.20093	-0.58304
C	-4.59331	1.25914	-0.08686
C	-5.87212	0.62486	-0.02698
H	-6.75377	1.21811	-0.28983
C	-6.00026	-0.68193	0.33815
H	-6.98145	-1.16545	0.35914
C	-4.85822	-1.41901	0.79804
C	-5.01464	-2.70951	1.35136
H	-6.00988	-3.16539	1.34100
C	-3.94932	-3.37509	1.92985
H	-4.09079	-4.36918	2.36459
C	-2.69421	-2.74701	1.99285
H	-1.85786	-3.23921	2.49787
C	-2.51273	-1.49672	1.42702
H	-1.53683	-1.01466	1.51801
C	-3.56458	-0.81800	0.77077
C	-3.40060	0.49872	0.16186
C	-2.14212	1.09766	-0.13877
C	-0.89804	0.39244	-0.39652
C	-0.75395	-0.88046	-1.01165
C	-1.74669	-1.62254	-1.69388
H	-2.74468	-1.19458	-1.80010
C	-1.44205	-2.85403	-2.23570
H	-2.20476	-3.42159	-2.77596
C	-0.13832	-3.36681	-2.09214
H	0.14108	-4.34342	-2.49279
C	0.81493	-2.59285	-1.44363
H	1.85025	-2.92292	-1.32054
C	0.36036	5.25424	-0.31870

Continued on next page

Table S12 – *Continued from previous page*

Symbol	X	Y	Z
H	0.07055	6.30544	-0.46034
H	1.05601	4.95538	-1.12332
H	0.86335	5.14614	0.65906
C	5.09120	2.13040	1.60594
H	4.57183	2.42528	2.53454
H	5.02604	2.98776	0.91312
H	6.14843	1.92825	1.83129
C	4.39181	0.94494	0.99921
C	5.11332	-0.23796	0.77878
H	6.16563	-0.22805	1.06805
C	4.61920	-1.43558	0.22324
C	5.55256	-2.60481	0.05850
H	5.14292	-3.47731	0.59695
H	6.56771	-2.39569	0.42671
H	5.60487	-2.88044	-1.00966

Table S13: XYZ coordinates for Λ_{Ir}-**A**

Symbol	X	Y	Z
C	-3.357765	-0.190134	2.509546
C	-2.421521	-0.239695	1.443327
N	-1.208755	0.453287	1.587165
C	-0.981599	1.173498	2.698072
C	-1.874691	1.260255	3.745856
C	-3.103902	0.543926	3.645090
C	-2.563324	-0.921648	0.196530
C	-1.523492	-0.733666	-0.794405
C	-1.626177	-1.302744	-2.066891
C	-2.703213	-2.121782	-2.371561
C	-3.701623	-2.386048	-1.422257
C	-3.607943	-1.808794	-0.172523
Ir	0.082839	0.244916	0.003419
N	1.366065	-0.200416	-1.561742
C	2.190294	-1.267375	-1.366150
C	3.082646	-1.650379	-2.378570
C	3.129782	-0.937979	-3.569644
C	2.284590	0.156825	-3.742932
C	1.420017	0.487302	-2.709657
C	2.042186	-1.902316	-0.056503

Continued on next page

Table S13 – *Continued from previous page*

Symbol	X	Y	Z
C	1.072960	-1.316834	0.811367
C	0.892861	-1.849105	2.096736
C	1.642078	-2.944810	2.499335
C	2.586737	-3.543117	1.669899
C	2.768354	-3.008666	0.402025
F	3.680933	-3.600814	-0.375016
F	1.462000	-3.453470	3.717631
F	-4.575346	-2.105437	0.713454
F	-2.803319	-2.690042	-3.584599
C	-0.698503	2.109207	-0.746087
N	-1.778684	2.601646	-1.395518
C	-1.698072	3.976813	-1.508742
C	-0.541131	4.363453	-0.912902
N	0.048488	3.207085	-0.454317
C	-2.900757	1.842944	-1.915651
C	1.249601	3.027757	0.269674
C	1.500858	1.697572	0.635836
C	2.684677	1.460455	1.348466
C	3.553387	2.505109	1.686749
C	3.263114	3.815673	1.308512
C	2.098735	4.086323	0.589287
H	4.465710	2.288624	2.252216
H	-0.109562	5.351749	-0.779389
H	-2.472122	4.562159	-2.000506
H	1.867821	5.110961	0.286050
H	3.941669	4.632978	1.569887
H	2.938308	0.442904	1.660519
H	-0.017189	1.687917	2.734388
H	-1.624575	1.858431	4.624351
H	-3.837797	0.577896	4.454988
H	-4.293261	-0.739822	2.409141
H	-4.538367	-3.048258	-1.655595
H	-0.859570	-1.139075	-2.830042
H	0.732060	1.331262	-2.793204
H	2.286572	0.749631	-4.659981
H	3.825422	-1.237562	-4.358414
H	3.735681	-2.506332	-2.222712
H	3.170115	-4.404965	1.997971
H	0.165445	-1.421038	2.790678
H	-2.581381	1.190429	-2.740871
H	-3.654213	2.553539	-2.284306

Continued on next page

Table S13 – *Continued from previous page*

Symbol	X	Y	Z
H	-3.342532	1.224558	-1.121753

Table S14: XYZ coordinates for (P,Λ_{Ir})-$\mathbf{A}^1$

Symbol	X	Y	Z
C	3.216065	0.466611	-0.058885
C	3.950807	0.159860	1.121182
C	5.314890	0.486178	1.178469
C	5.980631	1.096400	0.128113
C	5.238760	1.385847	-1.013770
C	3.888680	1.086778	-1.121469
C	3.188018	-0.490705	2.183251
N	1.878350	-0.719170	1.873665
C	1.076949	-1.311197	2.773500
C	1.511009	-1.709156	4.027577
C	2.845397	-1.485658	4.365019
C	3.681407	-0.877113	3.440580
Ir	1.248274	-0.104386	0.016054
C	0.770479	1.829751	0.596405
C	0.378170	2.671082	-0.483484
C	0.027003	4.004639	-0.217561
C	0.065499	4.546264	1.056832
C	0.477898	3.710962	2.091889
C	0.825684	2.384735	1.883779
C	0.395904	2.049823	-1.806751
N	0.784217	0.741920	-1.812759
C	0.864085	0.069903	-2.971891
C	0.558560	0.643611	-4.196155
C	0.151790	1.977220	-4.216084
C	0.071784	2.678315	-3.021314
F	-0.366002	4.822521	-1.204406
F	0.531356	4.219199	3.326147
F	6.040337	0.212636	2.271437
F	5.863328	1.974785	-2.036959
C	-0.630947	-0.972572	0.051016
N	-0.719258	-2.256589	-0.414496
C	-2.022411	-2.726062	-0.313046
C	-2.801406	-1.607628	0.120276
N	-1.889911	-0.592176	0.388758

Continued on next page

Table S14 – *Continued from previous page*

Symbol	X	Y	Z
C	-2.600914	-3.976987	-0.447235
C	-3.960627	-4.094981	-0.106008
C	-4.753601	-2.993060	0.260306
C	-4.203881	-1.667832	0.242019
C	-6.104877	-3.170273	0.706200
C	-6.863685	-2.090458	1.165789
C	-6.410311	-0.780652	1.014669
C	-5.120303	-0.550427	0.335491
C	-7.169749	0.349670	1.420031
C	-6.811451	1.614900	1.036704
C	-5.727477	1.833502	0.102526
C	-4.912445	0.723925	-0.298005
C	-5.492028	3.094311	-0.450354
C	-4.505610	3.289869	-1.435045
C	-3.766417	2.203425	-1.887122
C	-3.972835	0.934184	-1.332883
C	0.485563	-2.869770	-0.860366
C	0.542951	-4.147687	-1.415466
C	1.777797	-4.656811	-1.824482
C	2.945692	-3.901730	-1.690276
C	2.837499	-2.606297	-1.153941
C	1.626223	-2.045669	-0.732750
C	-2.162124	0.597082	1.175292
C	4.283980	-4.457282	-2.094257
H	-2.264996	1.488465	0.539350
H	-3.092606	0.444751	1.739268
H	-1.328563	0.753559	1.872405
H	-3.402254	0.086651	-1.720393
H	-3.020190	2.330817	-2.677132
H	-4.339533	4.287522	-1.851324
H	-6.100227	3.940386	-0.114995
H	-7.407727	2.477957	1.347574
H	-8.075532	0.186910	2.013136
H	-7.839541	-2.267251	1.628837
H	-6.499147	-4.187839	0.775947
H	-4.428173	-5.083129	-0.141257
H	-2.043739	-4.861482	-0.747596
H	-0.347915	-4.757361	-1.562102
H	1.821449	-5.660536	-2.260068
H	3.751435	-2.004817	-1.075222
H	4.178137	-5.322364	-2.770307

Continued on next page

Table S14 – *Continued from previous page*

Symbol	X	Y	Z
H	4.900172	-3.695904	-2.603359
H	4.858294	-4.796898	-1.211712
H	1.192293	-0.968501	-2.891428
H	0.642191	0.049966	-5.108988
H	-0.099478	2.472186	-5.158515
H	-0.237365	3.721019	-3.016046
H	-0.210802	5.586926	1.232511
H	1.133784	1.790542	2.749992
H	0.043247	-1.469363	2.462133
H	0.810516	-2.185042	4.717099
H	3.233986	-1.783642	5.342869
H	4.725769	-0.692548	3.681864
H	7.042179	1.337143	0.201134
H	3.375811	1.346394	-2.051890

Table S15: XYZ coordinates for (P,Δ_{Ir})-$\mathbf{A}^2$

Symbol	X	Y	Z
C	-3.209260	-0.320115	-0.063235
C	-3.638760	-0.515787	-1.406097
C	-4.991198	-0.797982	-1.654419
C	-5.931468	-0.894393	-0.641714
C	-5.484729	-0.700346	0.662157
C	-4.159710	-0.420480	0.962550
C	-2.596525	-0.396428	-2.422128
N	-1.361049	-0.096001	-1.926287
C	-0.320002	0.021028	-2.765477
C	-0.430129	-0.144377	-4.136770
C	-1.684857	-0.444218	-4.665891
C	-2.765761	-0.570088	-3.805941
Ir	-1.216186	0.127809	0.115562
C	-0.872185	-1.882278	0.490270
C	-0.932811	-2.221559	1.873193
C	-0.766273	-3.562512	2.254994
C	-0.543827	-4.576764	1.337930
C	-0.493264	-4.220174	-0.007135
C	-0.654241	-2.911338	-0.437186
C	-1.151732	-1.102394	2.788647
N	-1.278781	0.111617	2.177422

Continued on next page

Table S15 – *Continued from previous page*

Symbol	X	Y	Z
C	-1.477183	1.217226	2.912370
C	-1.560166	1.194603	4.295591
C	-1.433056	-0.033458	4.944053
C	-1.229046	-1.179823	4.189606
F	-0.817610	-3.922040	3.545257
F	-0.281229	-5.185924	-0.905349
F	-5.434449	-0.986418	-2.904793
F	-6.376166	-0.789688	1.652545
C	0.715582	0.880404	0.125037
C	-1.535299	2.205162	-0.138387
C	-0.358571	2.982917	-0.210183
C	-0.375980	4.348744	-0.490744
C	-1.604545	4.991380	-0.660384
C	-2.804325	4.282027	-0.562241
C	-2.737845	2.898972	-0.316196
C	-4.132677	4.972632	-0.709398
H	0.540164	4.926648	-0.611426
H	-1.618851	6.064102	-0.879817
H	-3.680741	2.340299	-0.269647
H	-4.024573	5.967235	-1.173723
H	-4.619325	5.117971	0.273458
H	-4.829607	4.379198	-1.326596
H	-1.577022	2.145677	2.346177
H	-1.723312	2.124749	4.844168
H	-1.493075	-0.099934	6.034096
H	-1.128235	-2.147128	4.676502
H	-0.418524	-5.610183	1.664340
H	-0.603471	-2.711807	-1.512082
H	0.641229	0.253999	-2.304677
H	0.454745	-0.036755	-4.767923
H	-1.821402	-0.581425	-5.742199
H	-3.753044	-0.807572	-4.195499
H	-6.976344	-1.114140	-0.865586
H	-3.889495	-0.279648	2.012809
N	0.836447	2.236504	-0.015045
C	2.161732	2.628314	0.115215
C	2.779672	3.856885	0.276705
H	2.239227	4.800810	0.256544
C	4.159156	3.848065	0.552851
H	4.663147	4.798894	0.748418
C	4.925849	2.669715	0.569140

Continued on next page

Table S15 – *Continued from previous page*

Symbol	X	Y	Z
C	6.305873	2.693123	0.959758
H	6.741398	3.647756	1.267553
C	7.046981	1.515303	1.072236
H	8.053459	1.542469	1.501422
C	6.535502	0.300717	0.614940
C	7.271996	-0.912247	0.663755
H	8.215901	-0.930000	1.218220
C	6.843762	-2.026306	-0.009631
H	7.423393	-2.953484	0.027695
C	5.701983	-1.968866	-0.897118
C	5.389336	-3.034654	-1.744296
H	5.982999	-3.952032	-1.680504
C	4.344327	-2.942939	-2.682425
H	4.120639	-3.791509	-3.335122
C	3.622031	-1.760710	-2.787197
H	2.830798	-1.659876	-3.536251
C	3.906202	-0.686834	-1.934146
H	3.353179	0.248379	-2.052920
C	4.908881	-0.775627	-0.942720
C	5.200770	0.285295	-0.015222
C	4.324716	1.408334	0.241870
C	2.914989	1.416128	0.197545
N	1.976454	0.391562	0.261594
C	2.245432	-0.969567	0.688161
H	1.491976	-1.260750	1.431358
H	2.204774	-1.672031	-0.157338
H	3.243771	-1.008648	1.143771

S3.2 Ir and Pt complexes spin unrestricted DFT optimizations for T_1

Table S16: XYZ coordinates *P*-**3a** from spin unrestricted geometry optimization.

Symbol	X	Y	Z
C	-2.05817	1.42850	-0.37177
C	-0.85181	0.71246	-0.73237
C	0.45501	1.30684	-0.45528
C	0.51846	2.66254	-0.32926
C	-0.66173	3.44152	-0.42606
C	-1.95383	2.83978	-0.40026
C	-3.28089	0.82228	-0.02626
C	-4.46995	1.60429	-0.13063
C	-4.35591	3.00037	-0.27799
C	-3.13488	3.61181	-0.34963
C	-3.42096	-0.56863	0.36505
C	-4.65663	-1.19088	0.08482
C	-5.80662	-0.39994	-0.18382
C	-5.72667	0.95921	-0.18565
C	-2.38305	-1.35584	0.95595
C	-2.44665	-2.75550	0.80403
C	-3.63616	-3.35769	0.33105
C	-4.73753	-2.60337	0.07352
C	-1.27871	-0.81396	1.71687
C	-0.16374	-1.64107	2.00283
C	-0.19280	-3.01382	1.63853
C	-1.31812	-3.56012	1.12078
C	-1.29686	0.47635	2.28410
C	-0.22507	0.96027	2.98904
C	0.91794	0.17312	3.17545
C	0.93594	-1.11198	2.70402
C	-0.79669	-0.51480	-1.41547
N	0.44161	-1.11958	-1.35963
C	0.60388	-2.33363	-1.87185
C	-0.42135	-3.01128	-2.50619
C	-1.65856	-2.38234	-2.65635
C	-1.84673	-1.13757	-2.11445
Pt	1.86836	-0.03109	-0.48886
O	3.18186	1.19969	0.39614
C	4.35961	0.85876	0.74017
C	4.96982	-0.37907	0.54471

Continued on next page

Table S16 – *Continued from previous page*

Symbol	X	Y	Z
C	4.42940	-1.49288	-0.10568
C	5.27270	-2.71857	-0.26357
O	-0.66510	4.78577	-0.44550
C	0.58687	5.46331	-0.46230
O	3.26667	-1.57551	-0.60400
C	5.13657	1.95395	1.39916
H	1.59356	-2.76217	-1.77029
H	-0.23673	-4.00224	-2.90386
H	-2.45980	-2.86890	-3.20254
H	-2.78599	-0.61023	-2.23248
H	1.47585	3.13694	-0.15091
H	-3.06939	4.69146	-0.42008
H	-5.26370	3.59493	-0.33353
H	-6.62032	1.56640	-0.29907
H	-6.75825	-0.90009	-0.33666
H	-5.68258	-3.07210	-0.18541
H	-3.67467	-4.43940	0.24029
H	-1.37554	-4.62680	0.92432
H	0.67743	-3.62978	1.84509
H	1.79488	-1.75158	2.88496
H	1.77213	0.57150	3.71338
H	-0.27042	1.95801	3.41405
H	-2.18141	1.09360	2.18545
H	4.50023	2.46298	2.12659
H	6.03779	1.58903	1.89097
H	5.42434	2.68750	0.63897
H	5.98294	-0.47923	0.91475
H	4.71174	-3.58812	0.08880
H	5.47987	-2.86861	-1.32772
H	6.21734	-2.65261	0.27533
H	0.33927	6.51831	-0.55721
H	1.12745	5.29977	0.47154
H	1.18398	5.14560	-1.31867

Table S17: XYZ coordinates *P*-**3c** from spin unrestricted geometry optimization.

Symbol	X	Y	Z
Pt	1.86562	-0.17551	-0.05674

Continued on next page

Table S17 – *Continued from previous page*

Symbol	X	Y	Z
N	0.50276	-1.36249	-0.89387
O	-0.83846	4.51796	-0.42455
O	3.11505	1.15474	0.77629
O	3.35527	-1.63139	-0.15292
C	0.38555	1.08977	-0.09678
C	0.40301	2.45399	-0.07571
H	1.34026	2.97257	0.08828
C	-0.79120	3.18204	-0.29072
C	-2.06700	2.53733	-0.27271
C	-3.26306	3.26731	-0.39436
H	-3.21958	4.33348	-0.58367
C	-4.47417	2.62669	-0.33446
H	-5.38973	3.18512	-0.50836
C	-4.56283	1.26228	-0.02492
C	-5.82075	0.60164	0.01223
H	-6.71004	1.17703	-0.22962
C	-5.91604	-0.71507	0.31504
H	-6.87777	-1.21916	0.30424
C	-4.76427	-1.44111	0.74663
C	-4.88457	-2.75713	1.22210
H	-5.85481	-3.24362	1.17346
C	-3.80983	-3.41062	1.77487
H	-3.92023	-4.42474	2.14671
C	-2.58790	-2.74503	1.90398
H	-1.75484	-3.23007	2.40295
C	-2.44265	-1.46759	1.41810
H	-1.49888	-0.95529	1.56628
C	-3.49954	-0.80615	0.77298
C	-3.36573	0.52356	0.20794
C	-2.13238	1.12974	-0.10527
C	-0.89446	0.43053	-0.37850
C	-0.76760	-0.82784	-0.99183
C	-1.77426	-1.55236	-1.65403
H	-2.74893	-1.09855	-1.78820
C	-1.50835	-2.80324	-2.14375
H	-2.27795	-3.36236	-2.66532
C	-0.23120	-3.34597	-1.97457
H	0.01640	-4.33765	-2.33432
C	0.74360	-2.58442	-1.36113
H	1.75642	-2.94830	-1.23720
C	0.38961	5.23823	-0.42771

Continued on next page

Table S17 – *Continued from previous page*

Symbol	X	Y	Z
H	0.11308	6.27341	-0.61496
H	1.04199	4.88040	-1.22614
H	0.88271	5.16338	0.54294
C	5.06038	2.09207	1.63061
H	4.54741	2.38401	2.55122
H	5.01045	2.93928	0.94060
H	6.10357	1.87252	1.85340
C	4.34708	0.92807	1.02312
C	5.04778	-0.25163	0.78723
H	6.09402	-0.25254	1.06655
C	4.54771	-1.43433	0.23267
C	5.46678	-2.60055	0.05970
H	5.05651	-3.45903	0.59893
H	6.47555	-2.39884	0.41789
H	5.50711	-2.86491	-1.00099

Table S18: XYZ coordinates (P,Δ_{Ir})-$\mathbf{A}^2$ from spin unrestricted geometry optimization.

Symbol	X	Y	Z
C	-3.13630	-0.31342	-0.08168
C	-3.53450	-0.52843	-1.42184
C	-4.86764	-0.82968	-1.68287
C	-5.81786	-0.93325	-0.69540
C	-5.39197	-0.72564	0.60041
C	-4.09183	-0.42286	0.92684
C	-2.48190	-0.38989	-2.41544
N	-1.27803	-0.02395	-1.90094
C	-0.23728	0.15095	-2.72233
C	-0.31234	-0.04554	-4.08140
C	-1.52501	-0.43983	-4.62177
C	-2.60888	-0.60685	-3.78693
Ir	-1.16847	0.17154	0.12845
C	-0.78676	-1.82146	0.44690
C	-0.87822	-2.20623	1.80701
C	-0.66425	-3.54006	2.14019
C	-0.37125	-4.50569	1.20654
C	-0.30454	-4.09835	-0.11076
C	-0.50648	-2.79830	-0.50694

Continued on next page

Table S18 – *Continued from previous page*

Symbol	X	Y	Z
C	-1.16270	-1.12952	2.74444
N	-1.27925	0.09716	2.16756
C	-1.52363	1.17182	2.92497
C	-1.67822	1.09723	4.29033
C	-1.57608	-0.14308	4.89808
C	-1.31506	-1.25437	4.12462
F	-0.73129	-3.95421	3.42648
F	-0.02171	-5.03763	-1.04270
F	-5.29699	-1.03029	-2.94953
F	-6.31302	-0.82399	1.58645
C	0.72342	0.93393	0.15942
C	-1.51538	2.22538	-0.09006
C	-0.36002	3.01924	-0.16843
C	-0.40326	4.38341	-0.39917
C	-1.64096	5.00327	-0.52704
C	-2.81787	4.27216	-0.43783
C	-2.72630	2.89350	-0.23155
C	-4.15436	4.93887	-0.54892
H	0.48828	4.98465	-0.50300
H	-1.67749	6.07431	-0.70628
H	-3.65001	2.31900	-0.18387
H	-4.06502	5.94794	-0.95815
H	-4.63173	5.01819	0.43396
H	-4.82767	4.36889	-1.19553
H	-1.60364	2.11245	2.39499
H	-1.87726	1.99845	4.85839
H	-1.69740	-0.24603	5.97127
H	-1.23358	-2.22557	4.58903
H	-0.21102	-5.53718	1.49790
H	-0.43896	-2.55906	-1.56455
H	0.68775	0.45956	-2.25634
H	0.56338	0.11480	-4.69944
H	-1.62795	-0.61217	-5.68778
H	-3.56039	-0.91134	-4.19609
H	-6.84896	-1.16929	-0.93112
H	-3.84249	-0.26932	1.97234
N	0.84482	2.28356	-0.01188
C	2.17168	2.66883	0.05410
C	2.81542	3.88295	0.09578
H	2.30338	4.82997	0.02755
C	4.20026	3.86967	0.28774

Continued on next page

Table S18 – *Continued from previous page*

Symbol	X	Y	Z
H	4.72159	4.81756	0.37840
C	4.94224	2.69030	0.34086
C	6.33985	2.69537	0.64422
H	6.82357	3.64724	0.83664
C	7.03926	1.50161	0.85774
H	8.04794	1.53847	1.26040
C	6.47379	0.28659	0.55519
C	7.12724	-0.96445	0.71477
H	8.05695	-1.00178	1.27636
C	6.63197	-2.08380	0.13466
H	7.14140	-3.03533	0.25442
C	5.50018	-2.02867	-0.77208
C	5.11287	-3.12820	-1.51235
H	5.63344	-4.07145	-1.37271
C	4.07027	-3.04451	-2.44480
H	3.78354	-3.92289	-3.01361
C	3.43773	-1.83483	-2.65732
H	2.66369	-1.75138	-3.41369
C	3.79942	-0.72014	-1.91159
H	3.33227	0.23794	-2.11244
C	4.79319	-0.80090	-0.92303
C	5.13118	0.27834	-0.06333
C	4.29822	1.43588	0.14649
C	2.91030	1.45948	0.16689
N	1.97721	0.44318	0.27803
C	2.25018	-0.88778	0.79757
H	1.51076	-1.11582	1.56102
H	2.20811	-1.64055	0.01113
H	3.23703	-0.88529	1.25641

Table S19: XYZ coordinates (P,Λ_{Ir})-**A**[1] from spin unrestricted geometry optimization.

Symbol	X	Y	Z
C	3.13224	0.47545	-0.11137
C	3.90559	0.22103	1.04608
C	5.25103	0.57531	1.04128
C	5.86502	1.16515	-0.03772
C	5.07848	1.40499	-1.14584

Continued on next page

Table S19 – *Continued from previous page*

Symbol	X	Y	Z
C	3.74468	1.07957	-1.20769
C	3.19686	-0.42199	2.14158
N	1.89924	-0.71753	1.86324
C	1.14868	-1.33254	2.78379
C	1.62092	-1.66424	4.03254
C	2.93349	-1.35092	4.34406
C	3.72089	-0.73459	3.39495
Ir	1.19731	-0.14083	0.04175
C	0.70043	1.76126	0.64569
C	0.27520	2.60175	-0.41135
C	-0.12377	3.90181	-0.11517
C	-0.09602	4.41913	1.15787
C	0.36665	3.58691	2.15775
C	0.76196	2.29039	1.93442
C	0.30110	2.00380	-1.73766
N	0.67026	0.69564	-1.75389
C	0.72249	0.03199	-2.91367
C	0.43052	0.62327	-4.12172
C	0.08202	1.96369	-4.13158
C	0.01725	2.65197	-2.93915
F	-0.57430	4.72802	-1.08672
F	0.41682	4.08693	3.41452
F	6.03215	0.34160	2.12049
F	5.66562	1.98636	-2.21726
C	-0.63772	-1.02665	0.12447
N	-0.72506	-2.31064	-0.33403
C	-2.03082	-2.76456	-0.27311
C	-2.79720	-1.64511	0.15134
N	-1.89208	-0.63766	0.43589
C	-2.63605	-3.98290	-0.47200
C	-4.00756	-4.07073	-0.20921
C	-4.78007	-2.96379	0.14115
C	-4.18286	-1.67313	0.19942
C	-6.15952	-3.08567	0.49727
C	-6.88278	-1.98917	0.98505
C	-6.36857	-0.71693	0.92269
C	-5.05480	-0.52888	0.27276
C	-7.05241	0.44901	1.36120
C	-6.61664	1.68157	1.00759
C	-5.52051	1.85874	0.07257
C	-4.78272	0.71662	-0.35367

Continued on next page

Table S19 – *Continued from previous page*

Symbol	X	Y	Z
C	-5.19450	3.10211	-0.43226
C	-4.18541	3.25351	-1.39292
C	-3.52619	2.13875	-1.87400
C	-3.82475	0.88056	-1.36792
C	0.48215	-2.91075	-0.78084
C	0.56192	-4.19771	-1.28477
C	1.79591	-4.67697	-1.70887
C	2.93403	-3.88517	-1.63295
C	2.80518	-2.58832	-1.13001
C	1.59424	-2.05727	-0.69991
C	-2.17350	0.53562	1.24683
C	4.27110	-4.41056	-2.05619
H	-2.24683	1.43627	0.63762
H	-3.10853	0.37260	1.78058
H	-1.36899	0.65553	1.96802
H	-3.32337	0.00858	-1.77440
H	-2.77571	2.23824	-2.65112
H	-3.94541	4.24164	-1.77138
H	-5.73749	3.97546	-0.08195
H	-7.14944	2.56781	1.33943
H	-7.95826	0.33459	1.95079
H	-7.87061	-2.14786	1.40904
H	-6.60766	-4.07372	0.50142
H	-4.49494	-5.03571	-0.30787
H	-2.10354	-4.86761	-0.78414
H	-0.29944	-4.84399	-1.37091
H	1.86074	-5.68677	-2.10479
H	3.69600	-1.96398	-1.08399
H	4.17069	-5.29098	-2.69543
H	4.83808	-3.65421	-2.60608
H	4.86740	-4.69870	-1.18336
H	1.02310	-1.00621	-2.84970
H	0.48889	0.03977	-5.03311
H	-0.13601	2.47238	-5.06473
H	-0.24342	3.69951	-2.93522
H	-0.41029	5.43691	1.35759
H	1.10467	1.69729	2.77833
H	0.13276	-1.56410	2.49336
H	0.96613	-2.15901	4.74035
H	3.34416	-1.58905	5.31951
H	4.74694	-0.48922	3.62446

Continued on next page

Table S19 – *Continued from previous page*

Symbol	X	Y	Z
H	6.91575	1.42911	-0.01041
H	3.19729	1.30027	-2.11917

Table S20: XYZ coordinates Λ_{Ir}-**A** from spin unrestricted geometry optimization.

Symbol	X	Y	Z
C	-3.35319	-0.19420	2.47946
C	-2.41621	-0.24770	1.42326
N	-1.20598	0.44585	1.57899
C	-0.99883	1.18877	2.67173
C	-1.89715	1.27830	3.70224
C	-3.11085	0.54685	3.60116
C	-2.54575	-0.91876	0.18267
C	-1.50065	-0.73207	-0.79468
C	-1.57866	-1.30602	-2.05532
C	-2.65716	-2.10133	-2.36907
C	-3.66644	-2.35969	-1.44915
C	-3.58019	-1.79298	-0.20691
Ir	0.08080	0.24840	0.01665
N	1.35430	-0.18131	-1.53481
C	2.16867	-1.25188	-1.35540
C	3.03969	-1.63361	-2.37181
C	3.08357	-0.91362	-3.54868
C	2.26422	0.19161	-3.69946
C	1.41669	0.52344	-2.66573
C	2.02776	-1.88564	-0.05124
C	1.06436	-1.30822	0.81522
C	0.88519	-1.83267	2.09467
C	1.64217	-2.90996	2.48786
C	2.58410	-3.50163	1.67111
C	2.75382	-2.97511	0.41040
F	3.67869	-3.57180	-0.36849
F	1.47048	-3.42500	3.72242
F	-4.57193	-2.09724	0.67324
F	-2.75019	-2.67191	-3.60287
C	-0.69224	2.08416	-0.72016
N	-1.77333	2.56901	-1.35637
C	-1.68455	3.93609	-1.49847

Continued on next page

Table S20 – *Continued from previous page*

Symbol	X	Y	Z
C	-0.52316	4.32508	-0.93459
N	0.06417	3.17802	-0.46356
C	-2.91920	1.81162	-1.82370
C	1.26278	2.99926	0.25464
C	1.49939	1.67814	0.62819
C	2.67899	1.43177	1.32750
C	3.55059	2.46568	1.65683
C	3.26996	3.76930	1.27635
C	2.11357	4.04686	0.56159
H	4.45627	2.24786	2.21616
H	-0.08918	5.30513	-0.83229
H	-2.45450	4.51307	-1.98436
H	1.89409	5.06455	0.25593
H	3.95101	4.57536	1.53059
H	2.92491	0.42120	1.63989
H	-0.05071	1.71475	2.70846
H	-1.66615	1.88728	4.56822
H	-3.84326	0.58186	4.40127
H	-4.28111	-0.74313	2.38796
H	-4.49837	-3.01131	-1.69536
H	-0.79867	-1.15922	-2.79675
H	0.75414	1.37660	-2.73355
H	2.27308	0.79221	-4.60131
H	3.75976	-1.21362	-4.34209
H	3.68003	-2.49314	-2.24100
H	3.17047	-4.35161	2.00097
H	0.15744	-1.41148	2.78063
H	-2.62752	1.13372	-2.62297
H	-3.65547	2.51859	-2.20174
H	-3.35295	1.25024	-0.99865

S3.3 Λ-$[Co(en)_3]^{3+}$

Table S21: XYZ coordinates for *lel*$_3$ Λ-$[Co(en)_3]^{3+}$ calculated at the B3LYP level of theory.

Symbol	X	Y	Z
Co	0.000000	0.000000	0.000000
N	1.501395	-0.731388	1.131190
N	-1.384098	0.934552	-1.131190
N	-0.117297	-1.665940	-1.131190
N	1.501395	0.731388	-1.131190
N	-0.117297	1.665940	1.131190
N	-1.384098	-0.934552	1.131190
C	-1.473659	-2.392605	0.749821
C	-1.335227	-2.472529	-0.749821
C	-1.335227	2.472529	0.749821
C	-1.473659	2.392605	-0.749821
C	2.808886	0.079924	-0.749821
C	2.808886	-0.079924	0.749821
H	2.868179	-0.885066	-1.257305
H	3.654196	0.682127	-1.093923
H	2.868179	0.885066	1.257305
H	3.654196	-0.682127	1.093923
H	-0.667600	2.926449	-1.257305
H	-2.417838	2.823563	-1.093923
H	-2.200579	2.041383	1.257305
H	-1.236359	3.505690	1.093923
H	-2.200579	-2.041383	-1.257305
H	-1.236359	-3.505690	-1.093923
H	-0.667600	-2.926449	1.257305
H	-2.417838	-2.823563	1.093923
H	-1.217875	-0.882155	2.143436
H	-2.319215	-0.525817	1.022348
H	-0.155031	-1.495788	-2.143436
H	0.704237	-2.271408	-1.022348
H	-0.155031	1.495788	2.143436
H	0.704237	2.271408	1.022348
H	-1.217875	0.882155	-2.143436
H	-2.319215	0.525817	-1.022348
H	1.372906	-0.613633	2.143436
H	1.614978	-1.745591	1.022348
H	1.372906	0.613633	-2.143436
H	1.614978	1.745591	-1.022348

Table S22: XYZ coordinates for *lel*$_3$ Λ-$[Co(en)_3]^{3+}$ calculated at the BP86 level of theory.

Symbol	X	Y	Z
Co	0.000000	0.000000	0.000000
N	1.494123	-0.732624	1.127868
N	-1.381533	0.927637	-1.127868
N	-0.112591	-1.660261	-1.127868
N	1.494123	0.732624	-1.127868
N	-0.112591	1.660261	1.127868
N	-1.381533	-0.927637	1.127868
C	-1.470551	-2.390399	0.750752
C	-1.334871	-2.468734	-0.750752
C	-1.334871	2.468734	0.750752
C	-1.470551	2.390399	-0.750752
C	2.805422	0.078335	-0.750752
C	2.805422	-0.078335	0.750752
H	2.865355	-0.893955	-1.262715
H	3.657328	0.684573	-1.100499
H	2.865355	0.893955	1.262715
H	3.657328	-0.684573	1.100499
H	-0.658489	2.928448	-1.262715
H	-2.421521	2.825053	-1.100499
H	-2.206865	2.034492	1.262715
H	-1.235807	3.509625	1.100499
H	-2.206865	-2.034492	-1.262715
H	-1.235807	-3.509625	-1.100499
H	-0.658489	-2.928448	1.262715
H	-2.421521	-2.825053	1.100499
H	-1.222487	-0.871791	2.149859
H	-2.324451	-0.516670	1.010312
H	-0.143750	-1.494600	-2.149859
H	0.714776	-2.271369	-1.010312
H	-0.143750	1.494600	2.149859
H	0.714776	2.271369	1.010312
H	-1.222487	0.871791	-2.149859
H	-2.324451	0.516670	-1.010312
H	1.366236	-0.622809	2.149859
H	1.609675	-1.754699	1.010312
H	1.366236	0.622809	-2.149859
H	1.609675	1.754699	-1.010312

Table S23: XYZ coordinates for *lel*$_3$ Λ-$[Co(en)_3]^{3+}$ calculated at the PBE0 level of theory.

Symbol	X	Y	Z
Co	0.000000	0.000000	0.000000
N	1.473378	-0.724707	1.119574
N	-1.364304	0.913629	-1.119574
N	-0.109074	-1.638336	-1.119574
N	1.473378	0.724707	-1.119574
N	-0.109074	1.638336	1.119574
N	-1.364304	-0.913629	1.119574
C	-1.447693	-2.358611	0.746333
C	-1.318770	-2.433044	-0.746333
C	-1.318770	2.433044	0.746333
C	-1.447693	2.358611	-0.746333
C	2.766463	0.074433	-0.746333
C	2.766463	-0.074433	0.746333
H	2.821465	-0.895887	-1.245496
H	3.617109	0.666253	-1.096636
H	2.821465	0.895887	1.245496
H	3.617109	-0.666253	1.096636
H	-0.634872	2.891404	-1.245496
H	-2.385547	2.799382	-1.096636
H	-2.186593	1.995517	1.245496
H	-1.231563	3.465635	1.096636
H	-2.186593	-1.995517	-1.245496
H	-1.231563	-3.465635	-1.096636
H	-0.634872	-2.891404	1.245496
H	-2.385547	-2.799382	1.096636
H	-1.203658	-0.858330	2.131165
H	-2.300669	-0.511936	1.008076
H	-0.141506	-1.471563	-2.131165
H	0.706985	-2.248406	-1.008076
H	-0.141506	1.471563	2.131165
H	0.706985	2.248406	1.008076
H	-1.203658	0.858330	-2.131165
H	-2.300669	0.511936	-1.008076
H	1.345164	-0.613233	2.131165
H	1.593684	-1.736470	1.008076
H	1.345164	0.613233	-2.131165
H	1.593684	1.736470	-1.008076

Table S24: XYZ coordinates for *lel*$_2$ *ob*$_1$ Λ-$[Co(en)_3]^{3+}$ calculated at the B3LYP level of theory.

Symbol	X	Y	Z
Co	0.000000	0.000000	0.000000
N	1.505032	-0.751011	1.113046
N	-1.383301	0.926103	-1.138590
N	-0.118163	-1.677114	-1.113851
N	1.505032	0.751011	-1.113046
N	-0.118163	1.677114	1.113851
N	-1.383301	-0.926103	1.138590
C	-1.503762	-2.381451	0.754030
C	-1.351214	-2.465053	-0.744119
C	-1.351214	2.465053	0.744119
C	-1.503762	2.381451	-0.754030
C	2.814156	0.657815	-0.368649
C	2.814156	-0.657815	0.368649
H	2.879513	1.501691	0.321515
H	3.657296	0.735970	-1.060577
H	2.879513	-1.501691	-0.321515
H	3.657296	-0.735970	1.060577
H	-0.717481	2.937459	-1.268473
H	-2.461145	2.790361	-1.088280
H	-2.204805	2.022875	1.261727
H	-1.263440	3.500207	1.085459
H	-2.204805	-2.022875	-1.261727
H	-1.263440	-3.500207	-1.085459
H	-0.717481	-2.937459	1.268473
H	-2.461145	-2.790361	1.088280
H	-1.198625	-0.878109	2.148121
H	-2.312655	-0.501050	1.045601
H	-0.134868	-1.526049	-2.129861
H	0.695241	-2.286981	-0.971958
H	-0.134868	1.526049	2.129861
H	0.695241	2.286981	0.971958
H	-1.198625	0.878109	-2.148121
H	-2.312655	0.501050	-1.045601
H	1.384792	-1.718432	1.435070
H	1.609243	-0.218233	1.984977
H	1.384792	1.718432	-1.435070
H	1.609243	0.218233	-1.984977

Table S25: XYZ coordinates for lel_1 ob_2 Λ-$[Co(en)_3]^{3+}$ calculated at the B3LYP level of theory.

Symbol	X	Y	Z
Co	0.000000	0.000000	0.000000
N	1.503502	-0.739693	1.121695
N	-0.089524	-1.673574	-1.119325
N	-1.413638	0.933882	-1.096818
N	1.503502	0.739693	-1.121695
N	-1.413638	-0.933882	1.096818
N	-0.089524	1.673574	1.119325
C	-0.822842	2.768609	0.385349
C	-1.969686	2.122747	-0.351088
C	-1.969686	-2.122747	0.351088
C	-0.822842	-2.768609	-0.385349
C	2.815990	0.093967	-0.748261
C	2.815990	-0.093967	0.748261
H	2.889251	-0.860632	-1.272822
H	3.656324	0.710268	-1.079473
H	2.889251	0.860632	1.272822
H	3.656324	-0.710268	1.079473
H	-0.125206	-3.247363	0.305082
H	-1.169097	-3.536140	-1.083043
H	-2.737926	-1.766298	-0.338020
H	-2.451317	-2.819660	1.042490
H	-2.737926	1.766298	0.338020
H	-2.451317	2.819660	-1.042490
H	-0.125206	3.247363	-0.305082
H	-1.169097	3.536140	1.083043
H	0.814047	2.045881	1.433465
H	-0.592472	1.494581	1.997091
H	-2.204026	0.350255	-1.393858
H	-1.020452	1.279867	-1.980558
H	-2.204026	-0.350255	1.393858
H	-1.020452	-1.279867	1.980558
H	0.814047	-2.045881	-1.433465
H	-0.592472	-1.494581	-1.997091
H	1.375019	-0.630390	2.135278
H	1.608414	-1.753015	0.997057
H	1.375019	0.630390	-2.135278
H	1.608414	1.753015	-0.997057

Table S26: XYZ coordinates for ob_3 Λ-$[Co(en)_3]^{3+}$ calculated at the B3LYP level of theory.

Symbol	X	Y	Z
Co	0.000000	0.000000	0.000000
N	-1.415176	-0.919958	1.103268
N	-1.415176	0.919958	-1.103268
N	1.504296	0.765600	-1.103268
N	-0.089119	-1.685558	-1.103268
N	-0.089119	1.685558	1.103268
N	1.504296	-0.765600	1.103268
C	2.817869	-0.658415	0.367898
C	2.817869	0.658415	-0.367898
C	-0.838730	2.769554	0.367898
C	-1.979139	2.111139	-0.367898
C	-0.838730	-2.769554	-0.367898
C	-1.979139	-2.111139	0.367898
H	-0.146346	-3.257053	0.321291
H	-1.193990	-3.533337	-1.065154
H	-2.747518	-1.755266	-0.321291
H	-2.462965	-2.800694	1.065154
H	-2.747518	1.755266	0.321291
H	-2.462965	2.800694	-1.065154
H	-0.146346	3.257053	-0.321291
H	-1.193990	3.533337	1.065154
H	2.893864	1.501787	0.321291
H	3.656955	0.732643	-1.065154
H	2.893864	-1.501787	-0.321291
H	3.656955	-0.732643	1.065154
H	1.384728	-1.742877	1.393182
H	1.599547	-0.259330	1.992266
H	1.384728	1.742877	-1.393182
H	1.599547	0.259330	-1.992266
H	0.817012	2.070648	1.393182
H	-0.575187	1.514913	1.992266
H	-2.201740	0.327771	-1.393182
H	-1.024360	1.255584	-1.992266
H	-2.201740	-0.327771	1.393182
H	-1.024360	-1.255584	1.992266
H	0.817012	-2.070648	-1.393182
H	-0.575187	-1.514913	-1.992266
